# Accelerated north–east shift of the global green wave trajectory

**DOI:** 10.1073/pnas.2515835123

**Published:** 2026-02-23

**Authors:** Miguel D. Mahecha, Guido Kraemer, Martin Reinhardt, David Montero, Fabian Gans, Ana Bastos, Hannes Feilhauer, Ida Flik, Chaonan Ji, Teja Kattenborn, Mirco Migliavacca, Milena Mönks, Johannes Quaas, Sebastian Sippel, Sophia Walther, Sebastian Wieneke, Christian Wirth, Gustau Camps-Valls

**Affiliations:** ^a^Institute for Earth System Science and Remote Sensing, Leipzig University, Leipzig 04109, Germany; ^b^Helmholtz Centre for Environmental Research (Helmholtz-Zentrum für Umweltforschungszentrum), Leipzig 04318, Germany; ^c^German Centre for Integrative Biodiversity Research (iDiv), Halle-Jena-Leipzig, Leipzig 04103, Germany; ^d^Center for Scalable Data Analytics and Artificial Intelligence (ScaDS.AI), Leipzig 04109, Germany; ^e^Max Planck Institute for Biogeochemistry, Jena 07701, Germany; ^f^Chair of Sensor-based Geoinformatics, University of Freiburg, Freiburg 79085, Germany; ^g^European Commission, Joint Research Centre, Ispra 21027, Italy; ^h^Leipzig Institute for Meteorology, Leipzig University, Leipzig 04109, Germany; ^i^Institute of Biology, Leipzig University, Leipzig 04109, Germany; ^j^Image Processing Laboratory, Universitat de València, Valencia 46010, Spain

**Keywords:** green wave trajectory, global greening, macrophenology, climate change impacts

## Abstract

Viewed from space, a “green wave” seasonally traverses Earth’s surface, from the north in boreal summer to the south in austral summer. This wave represents vegetation phenology, driven primarily by solar irradiation and modulated by climate variability and ecosystem dynamics. Despite its significance for multiple Earth system processes, we lack a unified metric to characterize and understand its dynamics. Here, we propose a concept to quantify global phenology by tracking the green wave’s centroid using satellite and Earth system model data. The resulting trajectory summarizes global phenological dynamics and directional trends. Earlier reports on global greening led us to hypothesize a rapidly northward shifting trajectory during boreal summer and a moderate southward shift during austral summer. Contrary to this expectation, we find that the centroid moves northward during both summer periods, with the austral summer shift consistently exceeding the boreal shift across datasets. As a consequence, the amplitude of the green wave trajectory is decreasing, a trend projected to intensify throughout this century. We also detect an accelerating eastward shift, a phenomenon not previously reported. Tracking the green wave’s centroid reveals how regionally changing land dynamics affect the global functioning of Earth’s terrestrial biosphere.

## Significance

Earth’s vegetation follows a rhythmic “green wave” that moves seasonally across the globe. It shapes the life cycles of organisms, biogeochemical cycles, and climate feedbacks, but until now, no intuitive metric existed to track its dynamics. We present a method to track the wave’s center of mass. This “flight path” of the green wave’s centroid reveals a measurable directional drift of ecosystem functioning: The green wave is shifting, with faster changes during Southern Hemisphere summers and an overall northeastward movement. This approach expresses planetary change caused by land use and climate change in kilometers over decades and offers a basis to monitor biosphere dynamics and their interaction with Earth system dynamics and human activity.

**Fig. 1.**
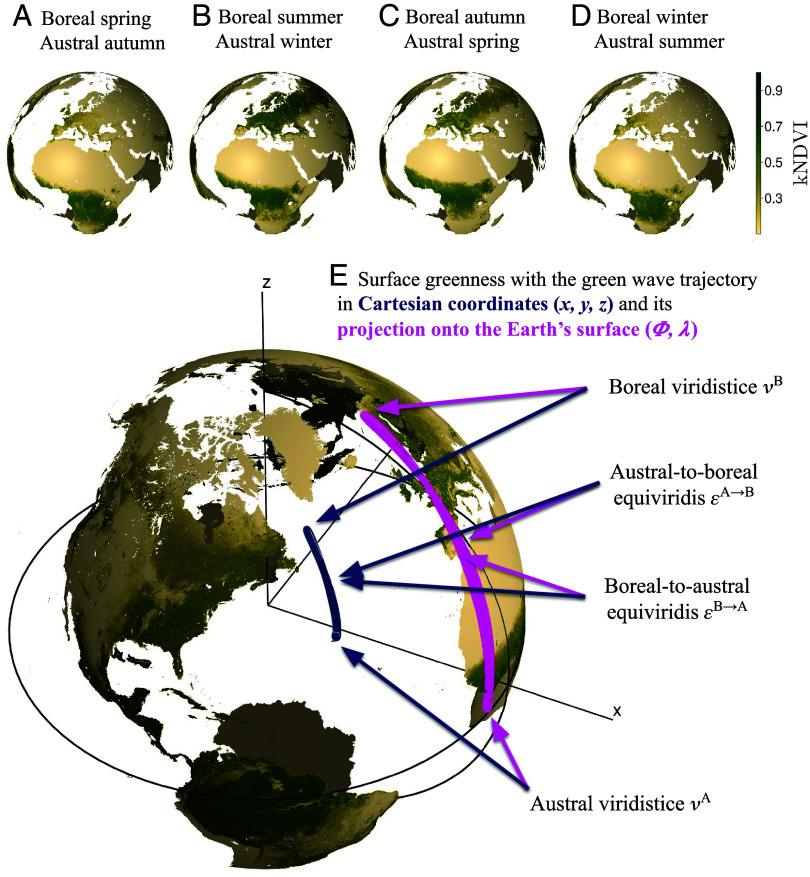
Mean seasonal cycle of a vegetation index observed from space and derived centroid trajectory. *Upper* panels show the “green wave” at stages corresponding to the 2023 boreal spring/autumn equinoxes and boreal summer/winter solstices on (*A*) March 20, (*B*) June 21, (*C*) September 23, and (*D*) December 21, using the kernel Normalized Difference Vegetation Index (kNDVI) derived from Moderate Resolution Imaging Spectroradiometer (MODIS) data. In (*E*), we present the three-dimensional trajectory of the centroid of green surfaces in a Cartesian coordinate system. The purple line is its projection to the two-dimensional projection onto the Earth’s surface.

Phenology, the study of seasonally recurrent patterns in plant organ development (1), is crucial for tracking the impacts of climate change on ecosystems (2, 3). At ecosystem scale, phenological stages such as spring green-up and senescence are globally observable via satellite remote sensing (2, 4–6). Observing phenology from space reveals a distinct “green wave” (7)—a seasonal north-to-south progression of vegetation development reflected in its greenness and leaf area index (Fig. 1). This macrophenological pattern (8) is driven primarily by seasonality but also encodes regional vegetation responses to intra- and interannual climate variability, as well as land-use and land-cover change (9, 10). By monitoring macrophenological dynamics, we can understand large-scale ecological processes, including land-atmosphere feedbacks (11), fire dynamics (12), large-scale drought impacts and recovery dynamics (13), and animal migration (14).

Phenology metrics are recognized as essential biodiversity variables (EBVs; 5, 15). Yet, unlike global metrics for temperature, radiative forcing, or sea ice extent—updated daily and widely accepted—there is no equivalent for global phenological dynamics. This gap limits our ability to monitor and integrate phenological changes with other long-term global trends. Most existing studies focus on specific subphenomena, such as regional trends in the timing of growing seasons, and provide derived summary statistics (e.g., refs. 16 and 17). While these approaches have significantly advanced our understanding of phenological shifts, they fall short of providing a comprehensive, universal metric for macrophenology.

We introduce a concept to quantify global phenology by tracking the trajectory of Earth’s green wave centroid over time. Leveraging global remote sensing data of vegetation properties—such as greenness indices and biophysical variables like the leaf area index (LAI)—this approach captures changes in global phenological dynamics over recent decades. When applied to Earth system models (ESMs), it offers insights into potential future shifts in global phenological dynamics and serves as a benchmark for evaluating and constraining land surface components in ESMs.

Given the widely reported global greening trend (16, 18–20) and the asymmetric distribution of landmasses, we expect the global green wave to exhibit a pronounced northward movement during the boreal summer in the Northern Hemisphere (NH) and a less pronounced southward shift during the austral summer in the Southern Hemisphere (SH). Consequently, we hypothesize an expansion in the amplitude of the green wave, with this trend expected to intensify under future climate scenarios.

## Results

### Green Wave Trajectory as an Indicator of Biosphere Dynamics.

To trace the centroid of the green wave at each moment in time, we calculate the center of mass of all land surfaces, weighted by greenness or LAI, in three spatial dimensions. Starting from the center of the Earth, the x-axis points toward the Equator–Greenwich meridian intersection (0°N, 0°E), the y-axis points toward the Equator–90°East intersection (0°N, 90°E), and the z-axis points toward the North Pole (90°N). See *Materials and Methods* for details on the computation. This Cartesian perspective enables a detailed analysis of the green wave’s centroid trajectory, including its velocity and acceleration at each point in time.

Using this approach, we quantify i) the annual amplitude of the centroid trajectory; ii) the exact timing of phenological phases, including spring, summer, autumn, and winter, for each hemisphere; and iii) long-term trends in these properties in terms of the green wave centroid positions at the phenological points, such as latitudinal and longitudinal drifts.

We interpret the green wave’s centroid trajectory as analogous to the solar year. A key moment in the solar year is the solstice—derived from the Latin words *sol* (sun) and *sistere* (stand still)—marking the day of highest hemispheric irradiation. Similarly, we define the viridistice (from *viridis*, meaning verdant) as the point in time when global vegetation greenness peaks in each hemisphere, denoted as $\nu_{\text{year}}^{A}$ for the austral viridistice and $\nu_{\text{year}}^{B}$ for the boreal viridistice per year. We determine the viridistice by identifying the seasonal minimum or maximum of the green wave centroid in the z-direction for the Northern and Southern Hemispheres, respectively (*Materials and Methods*).

During the solar year, the rate of change in day-night length peaks at the equinoxes. These are the two moments when the Earth’s axis is perpendicular to solar irradiation, resulting in nearly equal day and night lengths. At this time, the solar declination angle also reaches its maximum rate of change. Analogously, the points in the year where the green wave trajectory reaches its maximum speed represent the fastest transition of greenness between the hemispheres. We term the macrophenological equivalent of an astronomical equinox the “equiviridis,” denoted as $\varepsilon_{\text{year}}^{A\to B}$ for the austral-to-boreal summer transition and $\varepsilon_{\text{year}}^{B\to A}$ for the inverse. Fig. 1*E* illustrates an average green wave trajectory for the kernel Normalized Difference Vegetation Index (kNDVI; 21) in three dimensions (dark blue line). Its projection onto the Earth’s surface (purple line) lacks physical interpretation, yet geographic coordinates such as latitude ϕ and longitude λ provide an intuitive way to interpret and communicate the positioning of the green wave’s centroid trajectory.

### Characteristics of the Green Wave Trajectory.

Our definition of the green wave trajectory is straightforward and can be applied to any variable that captures the dynamics of terrestrial ecosystems, whether related to vegetation greenness or photosynthetic activity. Using the proposed analogy and its connection to solar declination, we aim to explore the mean seasonal trajectory in relation to the solar year. To this end, we first focus on the GIMMS LAI4g dataset (22), which is well suited for studying global greening and browning trends due to its extensive temporal coverage from 1982 to 2020 (23).

Fig. 2*A* illustrates the mean seasonal z-component of the GIMMS LAI4g trajectory, along with its velocity component (Fig. 2*B*) and overall trajectory speed (Fig. 2*C*). The figure also highlights the derived viridistices and equivirides. The boreal viridistice ($\nu^{B}$) corresponds to the seasonal maximum of the z-component, whereas the austral viridistice ($\nu^{A}$) marks its local minimum. The equivirides are identified as the points where the vertical velocity reaches a seasonal maximum ($\varepsilon^{A\to B}$) and a seasonal minimum ($\varepsilon^{B\to A}$), respectively. *SI Appendix*, Figs. S2–S25 show all three trajectory components (x, y, and z), their velocities, and the resulting timings across the different data products.

**Fig. 2.**
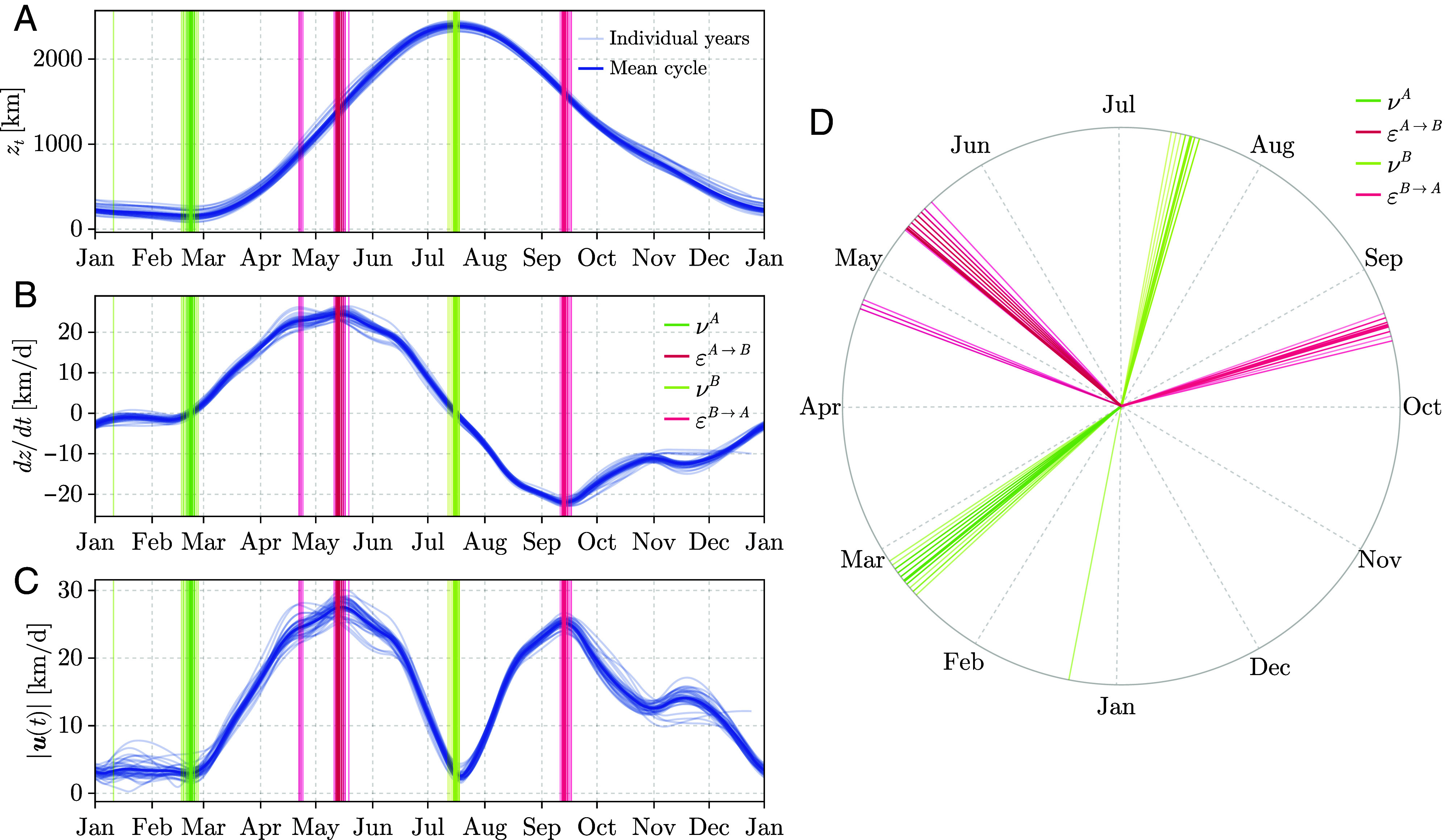
Seasonal cycles of the green wave centroid trajectory computed for leaf area index (LAI; 1982–2020). (*A*) The z-component of the 3D trajectory—representing the elevation above the equatorial plane (Fig. 1)—derived from GIMMS LAI4g (22). Vertical lines mark the austral viridistice ($\nu^{A}$), boreal spring equiviridis ($\varepsilon^{A\to B}$), boreal viridistice ($\nu^{B}$), and the austral spring equiviridis ($\varepsilon^{B\to A}$). (*B*) The corresponding z-component of velocity, which reaches a maximum at the boreal equiviridis and a minimum at the austral equiviridis. (*C*) The speed of the trajectory in all three spatial directions, with minima and maxima occurring close to—but not exactly at—the viridistices and equiviridis. The analogous x- and y-components are shown in *SI Appendix*, Figs. S3 and S4. (*D*) A circular representation of the yearly viridistice and equiviridis illustrating the seasonal asymmetry.

Displaying the four characteristic phenological moments in a circular plot reveals a clear seasonal asymmetry: Neither $\nu^{A}$ and $\nu^{B}$ nor $\varepsilon^{A\to B}$ and $\varepsilon^{B\to A}$ are positioned opposite each other (Fig. 2*D*). According to GIMMS LAI4g, the boreal viridistice $\nu^{B}$ occurs around July 16 (±1.44 d; circular SD, see *Materials and Methods*), almost one month after the boreal solstice (June 20/21). In contrast, the austral viridistice $\nu^{A}$ falls on February 21 (±7.32 d; circular SD), approximately two months after the austral solstice (December 20/21). This delay between the viridistice and the solstice is consistent across multiple remote sensing products (*SI Appendix*, Table S1). However, we find much larger interannual variability, as measured by circular SD, and larger differences among datasets in the austral viridistice (*SI Appendix*, Table S1 and Figs. S26–S33). Estimating $\nu^{A}$ is more challenging because the curvature of the z-component is flatter and often exhibits multiple local minima (Fig. 2).

Systematic differences also emerge between estimates derived from LAI- and greenness-based indicators (*SI Appendix*, Table S2). LAI products consistently show earlier boreal and austral viridistices than NDVI-based products, reflecting the fact that LAI captures canopy structure and leaf area, whereas NDVI tracks canopy greenness and may peak only after full chlorophyll saturation. To illustrate the generality of the centroid approach across ecophysiologically related variables—not to equate them—we also examined a wave metric derived from Gross Primary Production (GPP). GPP estimates from FLUXCOM-X-BASE (24) align more closely with LAI than with NDVI, particularly for the boreal viridistice and the upward equiviridis. This partial agreement is notable because GPP represents a different physiological process, and, based on first principles, peak GPP would be expected to lag behind peak leaf area due to the time required for leaf development (25–27). Spatial compensation effects may contribute to this alignment and merit further investigation.

**Fig. 3.**
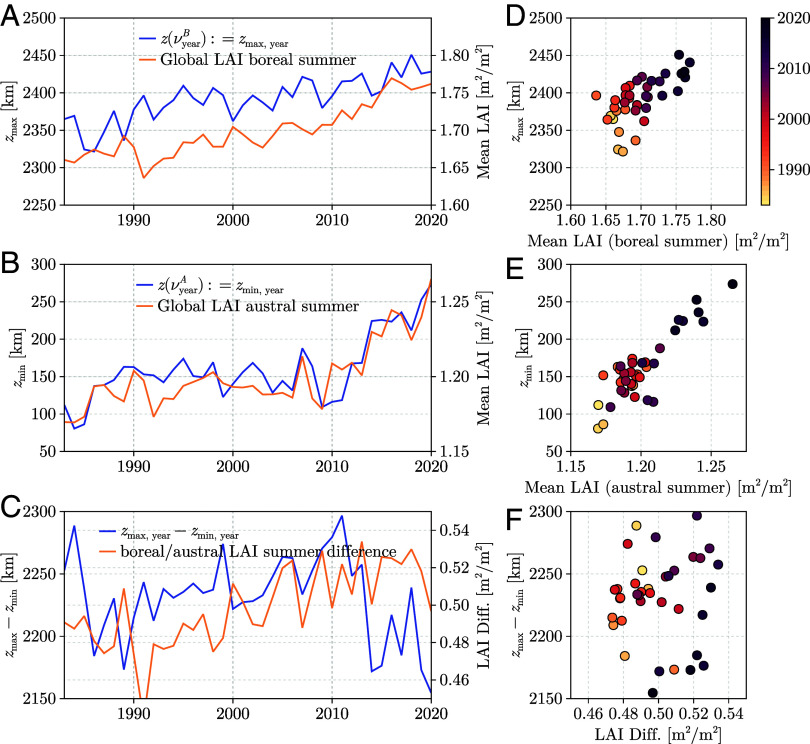
Shift of viridistice and global greening. (*A*) The boreal viridistice ($z_{\text{max}}$) exhibits a northward drift. (*B*) The austral viridistice ($z_{\text{min}}$) also shows a northward shift, particularly since 2010. This results in (*C*) an abrupt decrease in the spatial amplitude of the green wave. The relationships between the viridistice and average greening, computed globally for the boreal and austral summer months, are particularly strong for the austral summer, as shown in (*D*–*F*).

In terms of positioning, we find that the vertical position of the green wave centroid at the austral viridistice, $z(\nu^{A})$, lies slightly north of the equatorial plane at about 160 km, whereas the vertical position at the boreal viridistice, $z(\nu^{B})$, is around 2390 km north of the equatorial plane (GIMMS LAI4g; Fig. 3). This asymmetry between $z(\nu^{A})$ and $z(\nu^{B})$ reflects the unequal distribution of vegetated land masses on Earth—an effect that may also help explain the high interannual variability in the timing of $\nu^{A}$.

We also find a complex relationship between the equivirides (ε) and the equinoxes. While equinoxes are defined by equal day and night lengths (typically occurring around March 20 and September 22/23), the equivirides reflect the moments of maximal transition speed between hemispheric vegetation states—a process that appears to be strongly influenced by the unequal distribution of global green landmasses. Specifically, $\varepsilon^{A\to B}$ (the upward transition of the green wave toward the boreal spring) occurs on average on May 11 (±7.32 d; circular SD), nearly two months after the vernal equinox. In contrast, $\varepsilon^{B\to A}$ (the downward transition toward the austral spring) falls on September 14 (±1.3 d; circular SD), closely aligned with the autumnal equinox.

### Global Greening Induced Northward Shift.

Global trends in vegetation have been consistently documented across multiple satellite remote-sensing datasets, with the longest time series extending back to the 1980s (16, 18–20, 23). These data reveal that growing seasons are starting earlier (28, 29) and ending later (30), a key driver of global greening (31–33). This phenomenon, together with increasing seasonal amplitudes of vegetation growth due to rising CO_2_ fertilization, influences the annual integral of vegetation activity and derived remote-sensing indicators (16, 31, 34). The lengthening of the growing season has been most pronounced in the Northern Hemisphere, largely due to reduced temperature constraints in a warming climate (17, 35, 36). Regionally, land-use intensification—particularly for agriculture and silviculture in countries like China and India—has significantly contributed to greening trends (16, 19, 20, 37). Additionally, increased CO_2_ and nitrogen fertilization, which occur globally, suggest that similar shifts might be expected in the Southern Hemisphere (20, 38). Some studies suggest that global greening may be slowing (39) and could potentially reverse in the future as warming-induced heat and drought events increasingly limit growth (40).

These lines of evidence led us to hypothesize that the vertical positioning of the boreal viridistice would shift northward and the austral viridistice would shift southward. However, by fitting the dependency of the z-component on solar declination by decade with an analytic model identified via symbolic regression (Eq. **15**; see *Materials and Methods*), we find that the coefficients modeling the dependence on the solar declination angle δ remain constant, whereas the latitudinal shift parameter β moves northward. Specifically, we find:

$$\begin{array}{cc} \beta_{1982-1990} & =876\;\;\text{km},\\ \beta_{1991-2000} & =902\;\;\text{km},\\ \beta_{2001-2010} & =895\;\;\text{km},\\ \beta_{2011-2020} & =949\;\;\text{km}. \end{array}$$

This increase in β over time indicates a consistent northward shift of the green wave trajectory. In particular, the difference between $\beta_{1982-1990}$ and $\beta_{2011-2020}$ highlights the pronounced latitudinal movement.

Fig. 3*A* shows the time series of the vertical position of the trajectory at the boreal and austral viridistices. These positions correspond to $z(\nu_{\text{year}}^{B})$, the annual maximum of $z_{t}$, and $z(\nu_{\text{year}}^{A})$, the annual minimum of $z_{t}$, respectively, based on GIMMS LAI4g data from 1983 to 2021. Sen’s slope estimates indicate significant northward trends in the trajectory positioning at these time points (Mann–Kendall test): 2.0 km/year for $z(\nu_{\text{year}}^{B})$ and 2.4 km/year for $z(\nu_{\text{year}}^{A})$. More recently, between 2010 and 2020, these trends have accelerated to 3.3 km/year and 14.0 km/year, respectively, implying a decreasing amplitude of the green wave trajectory. These tendencies are consistent across other LAI products: GLASS-LAI (41) shows similar acceleration during 2010–2020, with trends of 4.5 km/year for $z(\nu_{\text{year}}^{B})$ and 10.02 km/year for $z(\nu_{\text{year}}^{A})$, while GLOBMAP-LAI (42) reports slopes of 6.4 km/year and 7.6 km/year, respectively. Although the absolute magnitudes differ among datasets, they consistently confirm that the austral viridistice exhibits a stronger northward shift than the boreal viridistice (for an overview of all trends see *SI Appendix*, Tables S3–S5).

The finding that trajectory positioning during the austral viridistice not only moves northward but does so more strongly than the boreal viridistice is unexpected, as we had anticipated a moderate southward movement mirroring the boreal viridistice. These effects are reproduced by Earth system models, as discussed later, and may result from extended growing seasons and milder winters in the NH, where larger landmasses allow even slight winter-season greening to offset the southward shift.

To compare the trends in trajectory positioning during viridistices with global greening, we computed mean global LAI values for consecutive equiviridis-defined seasonal windows, specifically $\left[\varepsilon_{\text{year}}^{A\to B},\varepsilon_{\text{year}}^{B\to A}\right]$ and $\left[\varepsilon_{\text{year}}^{B\to A},\varepsilon_{\text{year}+1}^{A\to B}\right]$. This analysis yields two global mean LAI time series: one for the boreal spring-to-autumn period ($\left[\varepsilon_{\text{year}}^{A\to B},\varepsilon_{\text{year}}^{B\to A}\right]$; Fig. 3*A*) and one for the austral spring-to-autumn period ($\left[\varepsilon_{\text{year}}^{B\to A},\varepsilon_{\text{year}+1}^{A\to B}\right]$; Fig. 3*B*). We find that the annual shift in the $z_{\min}$ position of the green wave centroid during the austral viridistice correlates strongly with the corresponding seasonal mean LAI (Fig. 3 *B* and *E*; R=0.84), particularly evident in its alignment with the breakpoint around 2009/2010 and smaller-scale anomalies. In contrast, the boreal shift in $z_{\max}$ shows only a moderate correlation with corresponding seasonal mean LAI (Fig. 3 *A* and *D*; R=0.66). The observed variability and recent decline in the amplitude of the green wave centroid trajectory are uncorrelated with changes in global seasonal LAI differences (Fig. 3 *C* and *F*).

### Unexpected Eastward Shift of the Global Green Wave.

**Fig. 4.**
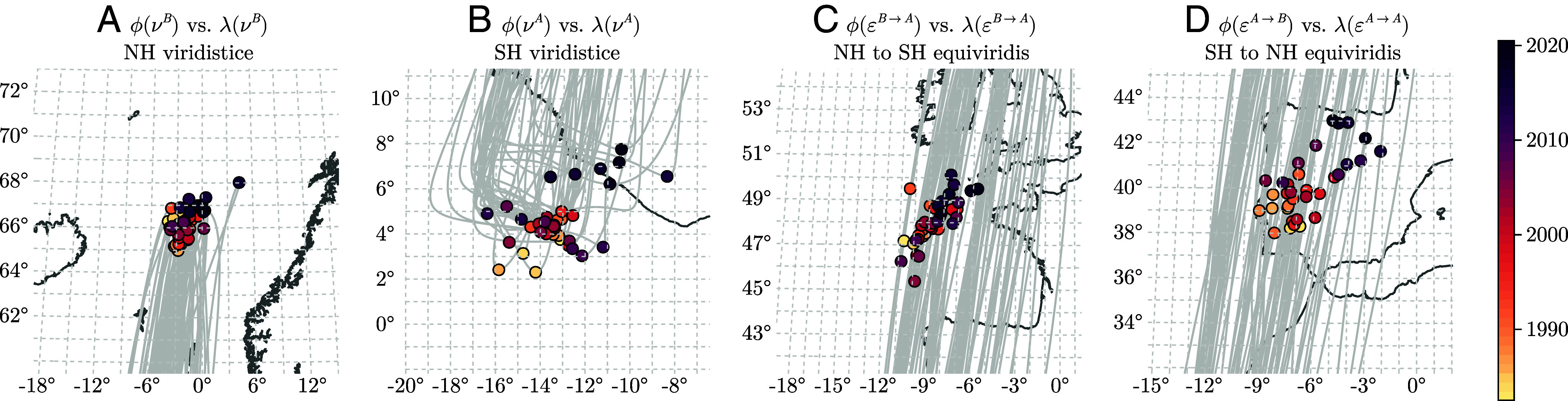
Mapping of the geographical coordinates corresponding to the two viridistices and equivirides onto the Earth’s surface. Thin gray lines represent the green wave trajectories projected onto the surface estimated using GIMMS LAI4g data (22) from 1982 to 2020. Panels emphasize the geographic positions of (*A*) the Northern Hemisphere viridistices, (*B*) the Southern Hemisphere viridistices, (*C*) the North-to-South equivirides, and (*D*) the South-to-North equivirides, shown as year-color-coded points along the green-wave trajectories.

Investigating the geographic projection of the green wave trajectory reveals an additional shift: The trajectory is shifting not only northward but also eastward, as clearly illustrated in Fig. 4. To interpret these trends, we estimated the slopes of the x, y, and z components during the viridistices using robust Sen’s slope statistics (*SI Appendix*, Fig. S34). These trends confirm a pronounced shift in positive z-direction, indicating a northward movement, and they reveal an equally strong trend in the y-direction, particularly for the austral viridistice. This suggests that the global green wave is shifting northward and eastward to a comparable degree. Data from 2010 onward reveal an accelerating northeastward movement of the trajectory during the austral viridistice.

**Fig. 5.**
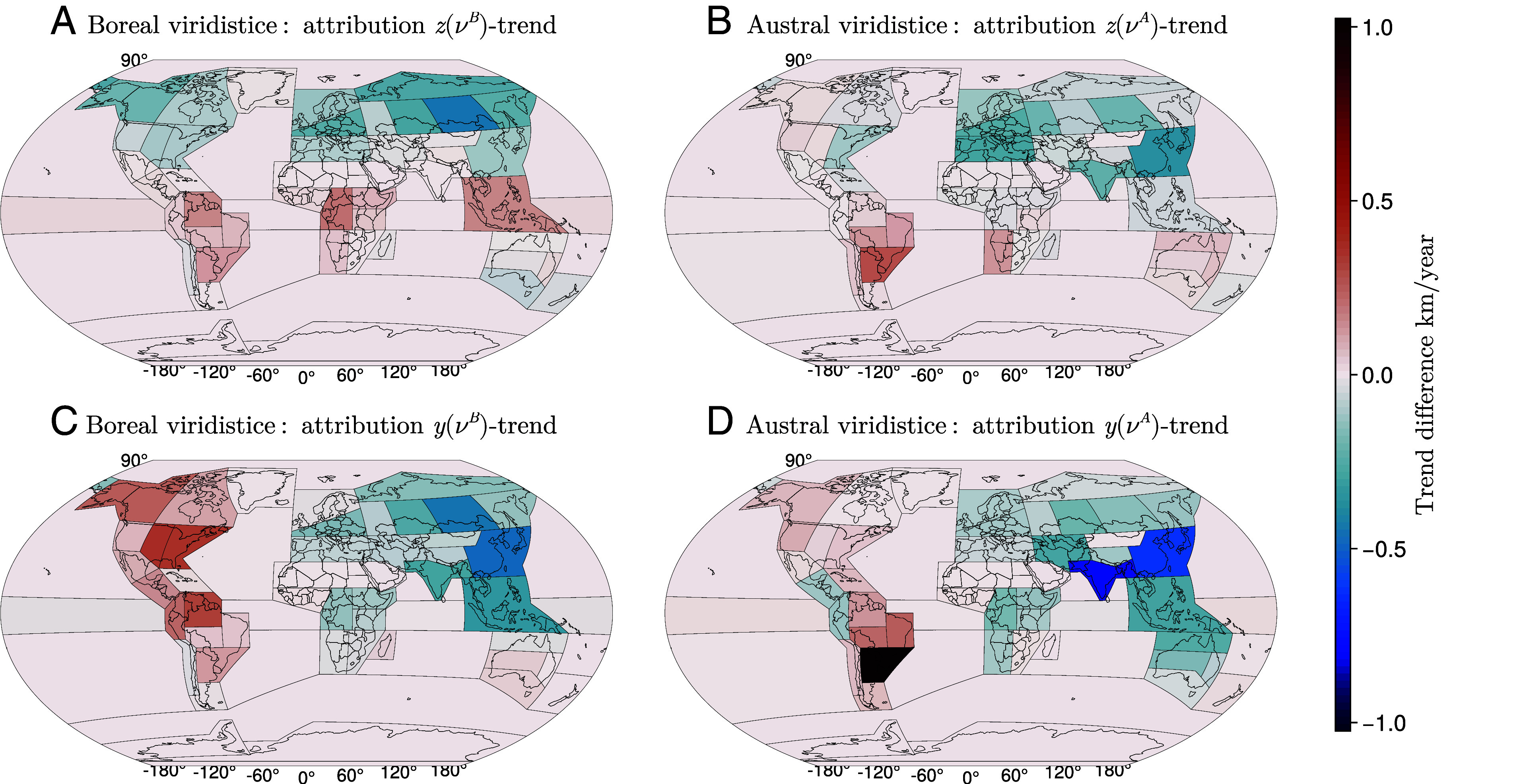
Trend difference without regional contributions. This analysis demonstrates how the observed trends in the trajectory positioning during the viridistice would change in z-direction (northward) and y-direction (eastward) if one of the regions remained constant in each of the four cases. (*A*) The boreal summer trend in $z(\nu_{\text{year}}^{B})$ is primarily driven by changes in the NH, particularly in Eastern and Western Siberia, with some moderate opposing contributions from the SH. (*B*) The austral summer northward shift in $z(\nu_{\text{year}}^{A})$ is primarily due to a pulling effect from Eastern Asia and the Mediterranean region, combined with a pushing effect from Southeastern South America and the South American Monsoon region. (*C*) The corresponding eastward shift of the trajectory ($y(\nu_{\text{year}}^{B})$) results from an antagonism between Eastern Asia and Eastern Siberia on one hand and Eastern and Central North America on the other. (*D*) The eastward shift in the trajectory at the austral viridistice ($y(\nu_{\text{year}}^{A})$) is mainly caused by opposing forces: an eastward pulling effect from Southern and Eastern Asia versus a pushing effect from Southeastern South America.

These findings are unexpected. The effect is likely linked to regional greening hotspots such as India, China, Europe, and—depending on the dataset—Russia (for a detailed analysis, see Cortes et al. 23). Such regional greening dynamics can shift the green wave trajectory toward these areas. To better understand these effects, we attribute the observed trends to contributions from different regions as defined by Iturbide et al. (43), the updated IPCC reference regions—a harmonized set of polygons designed for subcontinental analyses of observed and modeled climate change. Specifically, we iteratively recompute the global green wave trajectory while setting one region at a time to its mean seasonal cycle and leaving all others unchanged. This approach quantifies the contribution of each region to the trends in $z(\nu^{A})$ and $z(\nu^{B})$ (Fig. 5).

The results show that greenness dynamics in the NH are the dominant drivers of the overall northward shift in the green wave trajectory. Suppressing trends in these regions substantially weakens the global displacement (Fig. 5*A*). In the SH, regions such as South America and Oceania contribute to the northward movement of $z(\nu^{A})$, though the effect is less pronounced than in the NH (Fig. 5*B*). These findings emphasize regional contributions to global vegetation trends. The eastward component of the green wave shift is likewise shaped by opposing regional influences. During the boreal viridistice, the trajectory is pulled eastward by Eastern Asia and Eastern Siberia (Fig. 5*C*). During the austral viridistice, the eastward movement arises from antagonistic contributions: Southern and Eastern Asia pull the trajectory eastward, whereas Southeastern South America pushes it westward (Fig. 5*D*). Together, these regional contrasts determine the observed global eastward drift.

### Models Reproduce the North–East Shift and Project an Accelerated East Drift Throughout This Century.

**Fig. 6.**
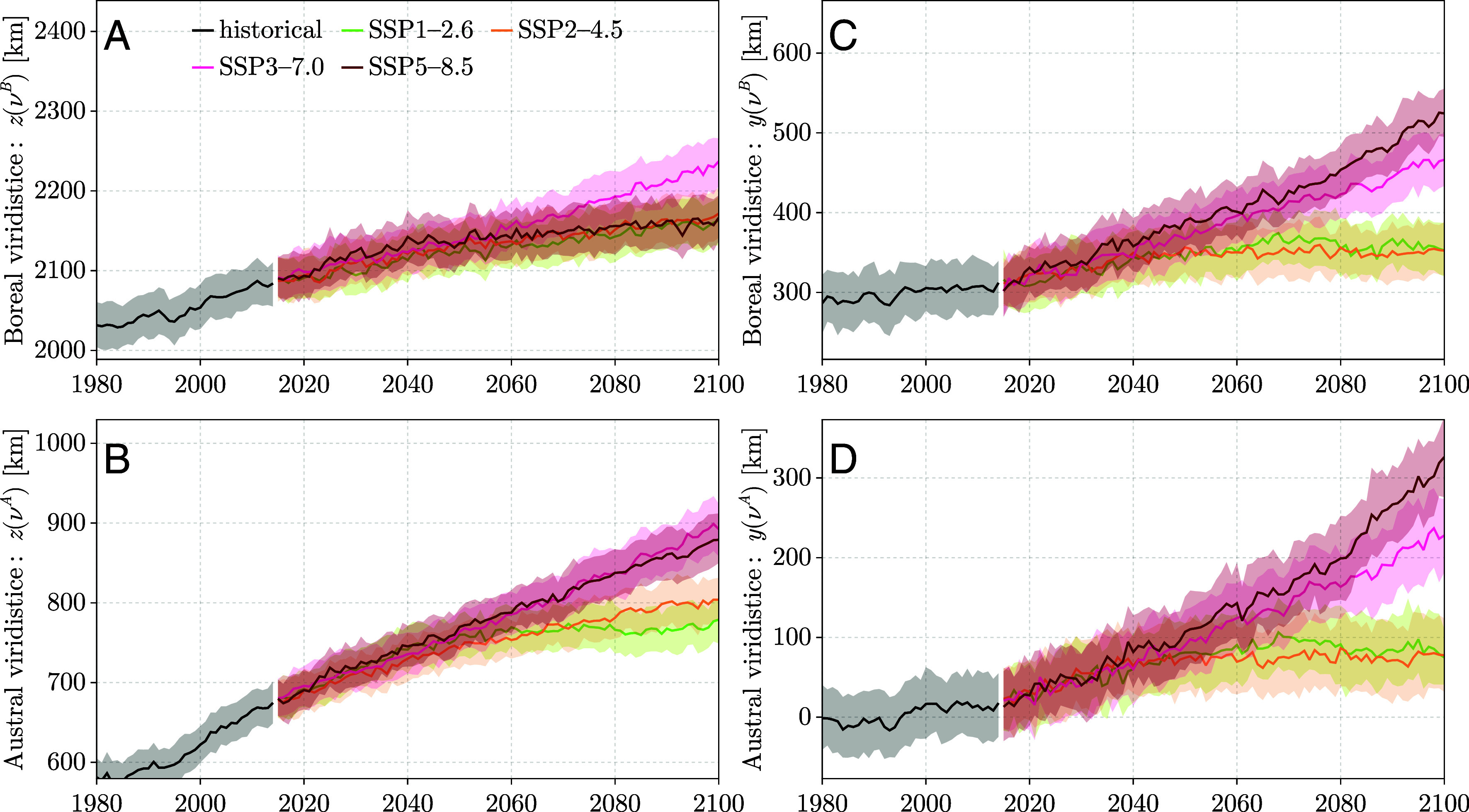
Projected shifts in viridistice positions reveal a northward and stronger eastward drift of the green wave trajectory. For each scenario, we generated a large synthetic ensemble by bootstrapping: In each bootstrap iteration, one ensemble member was randomly selected from each of the six retained CMIP6 models (ACCESS-ESM1-5, CanESM5, CESM2, FGOALS-g3, IPSL-CM6A-LR, MPI-ESM1-2-LR), and the viridistice positions were recomputed. The resulting distribution of bootstrap samples was used to derive the ensemble mean and the 2.5–97.5% quantile range. Panels show projected shifts for the historical period and four SSP scenarios: (*A*) northward shift of the boreal viridistice, $z(\nu^{B})$; (*B*) northward shift of the austral viridistice, $z(\nu^{A})$; (*C*) eastward shift of the boreal viridistice, $y(\nu^{B})$; and (*D*) eastward shift of the austral viridistice, $y(\nu^{A})$.

To assess how the observed changes in the green wave trajectory may evolve under different climate change scenarios, we analyzed LAI-derived trajectories from six Earth system models in the Coupled Model Intercomparison Project 6 (CMIP6). To ensure that models had sufficient process fidelity to encode the green wave, we applied a simple signal-to-noise criterion relating interannual variability to the green wave shift and retained only those with a detectable trend. A full description of this screening and the resulting model selection is provided in *SI Appendix* (results in *SI Appendix*, Table S1). Fig. 6 shows the ensemble averages of the retained models (ACCESS-ESM1-5, CanESM5, CESM2, FGOALS-g3, IPSL-CM6A-LR, and MPI-ESM1-2-LR), combining historical trends in viridistice positions (z(ν), y(ν)) with future projections based on the Shared Socioeconomic Pathways (SSP) scenarios. These scenarios include a sustainable development pathway (SSP1-2.6), a “Middle of the Road” pathway (SSP2-4.5), increasing regional rivalry (SSP3-7.0), and intensified fossil fuel exploitation (SSP5-8.5). The numbers (e.g., 2.6 to 8.5) represent the radiative forcing levels in W/m^2^ projected for 2100.

The model ensemble mean for the historical period generally reproduces the observed tendencies in green wave dynamics, even if absolute values differ. The models also show a northward shift in both boreal-summer viridistice positioning and an even stronger shift during the austral viridistice, implying a reduced north–south amplitude of the global green wave trajectory (Fig. 6). For the remainder of the century, the SSP3-7.0 scenario shows, on average, the strongest northward trend during the boreal viridistice, while SSP3-7.0 and SSP5-8.5 align in terms of their strong austral viridistice-position shifts. However, inspecting the models individually (*SI Appendix*, Figs. S37–S45), we observe substantial disagreement. For example, in ACCESS-ESM1-5 (*SI Appendix*, Fig. S37), scenario SSP5-8.5 consistently shows the strongest $z(\nu_{\text{year}}^{B})$ drift. Conversely, in some models, such as CanESM5 (*SI Appendix*, Fig. S38), CESM2 (*SI Appendix*, Fig. S39), or MPI-ESM1-2-LR (*SI Appendix*, Fig. S45), SSP1-2.6 exhibits the strongest $z(\nu_{\text{year}}^{B})$ shift. These discrepancies underscore substantial inter-model variability in green wave-trajectory projections, reflecting earlier findings on divergent land-carbon dynamics in CMIP6 models (44).

For the eastward shift, we find only a moderate trend in the historical simulations, followed by the onset of an even stronger displacement in the ensemble mean than in the northward direction (Fig. 6 *C* and *D*). In particular, the trajectory position during the austral viridistice shows a pronounced eastward shift, with scenario SSP5-8.5 clearly exceeding SSP3-7.0, both of which are above the more moderately shifting SSP2-4.5 and SSP1-2.6. Overall, in the high-forcing scenarios (SSP3-7.0 and SSP5-8.5), the eastward displacement is much stronger than the northward shift, whereas in the lower-forcing scenarios (SSP1-2.6 and SSP2-4.5), the northward component dominates. However, inspecting the trends on a model-by-model basis (*SI Appendix*, Figs. S37–S45) reveals substantial discrepancies among models.

As highlighted in earlier studies, the carbon cycle dynamics—tightly coupled to biosphere dynamics in CMIP models—are highly uncertain (45–47). Our results reinforce this view by showing that the proposed green wave centroid trajectory and its derived metrics, such as the trajectory position during viridistices, reveal substantial discrepancies among models. Rather than calling for better reproduction of local LAI or GPP fields, our metric provides an emergent, integrative benchmark of coupled land-biosphere-climate dynamics. Only models with sufficiently realistic terrestrial processes, human-induced land cover and land-use change, can reproduce the global trajectory in the historical domain, indicating that it may serve as a process-based emergent constraint for the next generation of Earth system models. The discrepancies among CMIP6 models in terms of signal-to-noise ratio and temporal dynamics (*SI Appendix*, Figs. S30–S36) further suggest that the green wave trajectory metric is highly sensitive to land-surface dynamics, making it a promising candidate for model calibration.

## Discussion

Our study reveals a significant ongoing shift in the global green wave toward the northeast. Even under the most optimistic future scenarios, the northward movement of the green wave persists, underscoring the impact of climate change on Earth’s ecosystems. Under scenarios of increased regional rivalry and intensified fossil fuel exploitation (SSP3-7.0 and SSP5-8.5), the shift in the green wave trajectory may become dominated by an accelerated eastward movement. Both the observed trends over the past decade and the simulated future projections show the most pronounced changes during austral summers, indicating shifting summer dynamics in the SH and altered winter dynamics in the NH. In particular, a distinct dipole is emerging between India and southeastern South America. These findings underscore the urgent need for continued research into the drivers of these shifts, their ecological and climatic consequences, and, in particular, for improving the representation of vegetation dynamics in Earth system models.

The observational results presented here are consistent across data products, both in the timing of the seasonal green wave and in its long-term dynamics. Nonetheless, all these data products rely on remote-sensing retrievals and postprocessing routines and therefore inherit their limitations, which are not equally reliable at the local scale. For example, NDVI is known to perform poorly when the signal-to-noise ratio is low, such as in some evergreen needle-leaf forests (22, 41, 48). Similar biome-specific issues affect other vegetation types, including known limitations in LAI retrievals (49). However, these effects recur systematically each year and partially compensate across space, and thus may influence the detailed shape of the green wave trajectory but should have limited influence on its long-term change. The fact that we find highly consistent trends and interannual variability across multiple, independently derived datasets-and even across variables representing different biophysical and biogeochemical processes-gives us confidence that the green wave shift we report reflects a robust, large-scale phenomenon in the terrestrial biosphere. The emergence of the green wave in most CMIP6 models, which were not explicitly designed to represent this process, provides an additional and independent indication of the robustness of our findings.

From a conceptual point of view, our study introduces a metric for tracing macrophenological dynamics: the global “green wave trajectory.” This metric represents the three-dimensional (3D) path of the center of mass of green surfaces, capturing both seasonal patterns and long-term trends such as global greening. Existing approaches typically rely on interpreting local phenological metrics (6, 9, 28, 32, 50). These studies have greatly advanced our understanding of how local ecosystems respond to human and natural pressures. Our approach complements these efforts by providing a global directional drift of ecosystem functioning. It allows us to read climate-change impacts like a compass and quantify a measurable spatial relocation of where the planet’s greenness is centered. By tracking the green wave trajectory, we can express planetary change in concrete spatial units, enabling us to describe large-scale biosphere shifts quantitatively over time.

While new to Earth observation, similar concepts have been applied in other fields, such as determining the center of human population (51) and tracking the center of mass of the global economy (52). A particularly relevant feature of our metric is its ability to compare variables of different physical units. For instance, we observe remarkable consistency in interannual variability across products, even when they represent different yet coupled biophysical and biogeochemical processes (LAI, NDVI, GPP; *SI Appendix*, Fig. S4). In our framework, the dynamics of variables are mapped into the same coordinate system, allowing for a direct comparison of their respective global trajectories. We argue that this level of abstraction can offer substantial advantages for comparing processes and model evaluation. The macroscopic metric can potentially be used as a benchmark for Earth system models (53) or as an emergent constraint (54, 55).

As global climate change accelerates, the ability to monitor and understand these changes through robust, unified metrics is critical for effective adaptation and mitigation strategies. Initially developed to quantify global phenology, the green wave centroid framework offers a versatile and consistent basis for analyzing many other Earth-subsystem dynamics. We envisage a “blue wave” summarizing terrestrial water storage, “white waves” for snow and ice coverage, or a “red wave” for fire patterns. Likewise, it could be applied to ocean systems, offering insights into phytoplankton cycles (“turquoise wave”), or other domains in which global or regional shifts in spatial centroids may signal critical environmental changes. Given antecedent analyses of socioeconomic centroids, the metric is well suited for assessing human-environment linkages at regional scales. Trends in human-made mass (56) or built-up area driven by urban expansion or road-network growth, could be examined in the same vein as “gray wave” and related to their impacts on the biosphere’s green wave. More broadly, integrating such metrics into a holistic, multivariate framework would enable a more comprehensive understanding and modeling of Earth system domains, their couplings, and their interactions with human activity.

## Materials and Methods

### Trajectory of the Green Wave.

In the following, we describe how to derive the trajectory of the green wave’s center of mass. This approach can be applied to any spatiotemporal dataset over the Earth’s surface at both global and regional scales. The objective is to estimate the trajectory of the center of mass of the terrestrial surface, $c_{t}$, where “mass” refers to the weights assigned by a spectral index or a biophysical variable that indicates global phenology at time t. Candidate variables for computing the “Center of Vegetation Activity” include the leaf area index (LAI), the Normalized Difference Vegetation Index (NDVI) or its nonlinear extension, the kernel NDVI (KNDVI) (21), and Gross Primary Production (GPP). However, other relevant indices may also be considered. We assume that these variables are globally curated as data cubes $\rho_{\lambda_{i},\phi_{i},t}$ (57, 58), where $\lambda_{i}$ and $\phi_{i}$ represent the longitude and latitude of grid cell i=1,…,N denoting the spatial grid cells, and t=1,…,T representing the time steps. The first step in computing the 3D trajectory of the centroid $c_{t}$ is to transform the spherical coordinates of each grid cell $\left(\lambda_{i},\phi_{i}\right)$ into a Cartesian coordinate system:

[1] $$\begin{array}{cc} X_{i} & =\left(v_{i}+H\right)\cos\phi_{i}\cos\lambda_{i},\\ Y_{i} & =\left(v_{i}+H\right)\cos\phi_{i}\sin\lambda_{i},\\ Z_{i} & =\left(\left(1-e^{2}\right)v_{i}+H\right)\sin\phi_{i}, \end{array}$$

where

$$\begin{array}{c} v_{i}=\frac{a}{\sqrt{1-e^{2}\sin^{2}\phi_{i}}}\;\;\text{and}\;e^{2}=\frac{a^{2}-b^{2}}{a^{2}}, \end{array}$$

with parameters a=6,378.14 km and b=6,356.75 km representing the semimajor and semiminor axes of the WGS84 ellipsoid, and H denoting the height above the ellipsoid. All Cartesian coordinates ($X_{i},Y_{i},Z_{i}$) are therefore expressed in kilometers.

We then estimate the centroid for each time step by computing the weighted averages of the coordinates for all grid cells:

[2] $$\begin{array}{c} \begin{array}{cc} c_{t} & :={\left({\overset{¯}{x}}_{t},{\overset{¯}{y}}_{t},{\overset{¯}{z}}_{t}\right)}^{⊤}\\ & ={\left(\frac{\sum_{i=1}^{N}w_{i,t}X_{i}}{\sum_{i=1}^{N}w_{i,t}},\frac{\sum_{i=1}^{N}w_{i,t}Y_{i}}{\sum_{i=1}^{N}w_{i,t}},\frac{\sum_{i=1}^{N}w_{i,t}Z_{i}}{\sum_{i=1}^{N}w_{i,t}}\right)}^{⊤}. \end{array} \end{array}$$

If we collect the spatial position vectors $p_{i}={\left(X_{i},Y_{i},Z_{i}\right)}^{⊤}$ as columns of a matrix $P\in R^{3\times N}$, and have the vector of weights $w_{t}={\left(w_{1,t},\ldots ,w_{N,t}\right)}^{⊤}\in R^{N}$, then we can express the estimation of the centroid of the trajectory for each time point as

[3] $$\begin{array}{c} c_{t}=\frac{P\;w_{t}}{1^{⊤}w_{t}}\;\in \;R^{3}, \end{array}$$

where 1 is an N-vector of ones. Note that the weight $w_{i,t}$ for each spatial grid cell i and time point t is computed as the product of the variable of interest $\rho_{\lambda_{i},\phi_{i},t}$ and the corresponding area weight:

[4] $$\begin{array}{c} w_{i,t}=A_{\lambda_{i},\phi_{i}}\cdot \rho_{\lambda_{i},\phi_{i},t}\propto \;\cos\phi_{i}\cdot \rho_{\lambda_{i},\phi_{i},t}. \end{array}$$

Here, $A_{\lambda_{i},\phi_{i}}$ denotes the physical surface area of grid cell i on the WGS84 ellipsoid. For regular latitude-longitude grids, $A_{\lambda_{i},\phi_{i}}$ can be approximated by a factor proportional to $\cos\phi_{i}$. If desired, the resulting trajectory $c_{t}$ can be projected back into spherical coordinates $\left(\phi_{t},\lambda_{t}\right)$ for mapping the trajectory onto Earth’s surface.

### Smoothing.

To robustly estimate the trajectory and the derived viridistice, we applied the Schmid–Rath–Diebold (SRD) filter (59), an improved version of the Savitzky–Golay filter (60), configured with half-kernel width m=80 d and polynomial degree n=2, which preserves seasonal peaks while suppressing short-term variability. Each component of the trajectory $c_{t}$ is smoothed using the SRD filter. For an odd kernel length 2m+1 with coefficients ${\left\{K_{k}\right\}}_{k=-m}^{m}$, we obtain

[5] $$\begin{array}{cc} {\tilde{c}}_{t} & ={\left({\tilde{x}}_{t},{\tilde{y}}_{t},{\tilde{z}}_{t}\right)}^{⊤}\\ & ={\left(\sum_{k=-m}^{m}K_{k}\;{\overset{¯}{x}}_{t-k},\;\sum_{k=-m}^{m}K_{k}\;{\overset{¯}{y}}_{t-k},\;\sum_{k=-m}^{m}K_{k}\;{\overset{¯}{z}}_{t-k}\right)}^{⊤}. \end{array}$$

The smoothed series (e.g., $\left\{{\tilde{z}}_{t}\right\}$) is interpolated to daily resolution using cubic splines with smoothing factor s=0, providing exact interpolation to ${\tilde{z}}_{t}$ at the original sampling times (e.g. Fig. 2*A*) and smooth first and second derivatives (Fig. 2 *B* and *C*). We denote the resulting continuous functions by x(t), y(t), and z(t).

### Viridistice and Equiviridis.

The viridistice, $\nu_{\text{year}}$, denotes the time point in a year when the z-component of the trajectory reaches its hemispheric extrema. The austral summer viridistice is thus defined as

[6] $$\begin{array}{c} \nu_{\text{year}}^{A}=\;\arg\min_{t\in \;\text{year}}z\left(t\right), \end{array}$$

and the boreal summer viridistice as

[7] $$\begin{array}{c} \nu_{\text{year}}^{B}=\;\arg\max_{t\in \;\text{year}}z\left(t\right). \end{array}$$

The corresponding spatial coordinates at these times are ${\left(x\left(\nu \right),y\left(\nu \right),z\left(\nu \right)\right)}^{⊤}$, or in spherical form (λ(ν),ϕ(ν)).

The equiviridis, $\varepsilon_{\text{year}}$, denotes the moment of maximum vertical transition speed during the annual cycle. The upward transition toward positive z marks the onset of boreal spring:

[8] $$\begin{array}{c} \varepsilon_{\text{year}}^{A\to B}=\;\arg\max_{t\in \;\text{year}}\left\{\frac{dz(t)}{\mathrm{dt}}\right\}, \end{array}$$

while the moment of maximum negative z-velocity indicates the onset of austral spring:

[9] $$\begin{array}{c} \varepsilon_{\text{year}}^{B\to A}=\;\arg\min_{t\in \;\text{year}}\left\{\frac{dz(t)}{\mathrm{dt}}\right\}. \end{array}$$

Because the timing of the viridistice $\nu_{\text{year}}$ may occur near the turn of the calendar year, particularly in the Southern Hemisphere, we analyze means and interannual statistics using circular statistics (61, 62). The DOY associated with $\nu_{\text{year}}$ is transformed into an angular variable:

[10] $$\begin{array}{c} \theta_{\text{year}}=2\pi \frac{\text{DOY}(\nu_{\text{year}})}{365}. \end{array}$$

The circular mean is then computed as

[11] $$\begin{array}{c} C=\left\langle \cos\theta_{\text{year}}\right\rangle ,\;S=\left\langle \sin\theta_{\text{year}}\right\rangle , \end{array}$$

[12] $$\begin{array}{c} \mu =\text{atan2}\left(S,C\right),\;\bar{\nu }=365\;\frac{\mu }{2\pi }. \end{array}$$

Circular variability is quantified from the mean resultant length:

[13] $$\begin{array}{c} R=\sqrt{C^{2}+S^{2}}, \end{array}$$

[14] $$\begin{array}{c} \sigma_{\text{circ}}=\sqrt{-2\ln R}, \end{array}$$

and $\sigma_{\text{circ}}$ is expressed in days by multiplying with 365/(2π).

### Symbolic Regression.

Returning to the assumption that the green wave trajectory can be expressed as a function of the solar declination angle, δ, we can identify an analytic model for the z-position (in km). Advances in data-driven methodologies now make it possible to discover such relationships directly from data (63). A popular approach in this context is symbolic regression (64–66). Using a multipopulation evolutionary algorithm method proposed by Cranmer (67), we identified the following model that accurately represents the green wave trajectory of LAI (from GIMMS LAI4g):

[15] $$\begin{array}{c} z\left(t\right)=0.67\cdot \delta_{\tau }{\left(t\right)}^{2}+46.71\cdot \delta_{\tau }\left(t\right)+\beta , \end{array}$$

where $\delta_{\tau }\left(t\right)$ is the solar declination angle at time t, incorporating a fixed time lag τ. The lag, τ=−32d, reflects the time delay between the solstice and viridistice and is predetermined through cross-correlation. The time-lagged solar declination angle is defined as

[16] $$\begin{array}{c} \delta_{\tau }\left(t\right)=23.44^{{}^{\circ}}\cdot \sin\left(360^{{}^{\circ}}\cdot \frac{{\text{DOY}}_{t}+\tau -81}{365}\right), \end{array}$$

where ${\text{DOY}}_{t}$ represents the day of the year corresponding to time t. The offset β represents the baseline of the z-component of the green wave trajectory when $\delta =0^{{}^{\circ}}$ has an average value of β=9 km. Eq. **15** fits the mean seasonal cycle of z(t) with a Nash-Sutcliffe Efficiency of NSE=0.67 and a Kling-Gupta Efficiency of KGE=0.84, indicating that the solar declination angle can effectively describe the green wave dynamics.

## Supplementary Material

Appendix 01 (PDF)

## Data Availability

All datasets used in this study are publicly available, and their postprocessing procedures are documented in detail in *SI Appendix*. The full codebase, including scripts for data analysis and figure generation, is available here (mainly in the Julia language): https://doi.org/10.5281/zenodo.18430946 (68).
