# Supplementary material for: Accelerated north–east shift of the global green wave trajectory
Source: Proc Natl Acad Sci U S A. 2026 Feb 23;123(9):e2515835123. doi: 10.1073/pnas.2515835123 (PMC12956857; doi:10.1073/pnas.2515835123)
Supplement: Supplementary file 1 — Appendix 01 (PDF) [file pnas.2515835123.sapp.pdf]

Supporting Information for

Accelerated North-East Shift of the Global Green Wave Trajectory

Miguel D. Mahecha et al.

Corresponding Author: Miguel Mahecha
E-mail: miguel.mahecha@uni-leipzig.de

This PDF file includes:

Supporting text
Figs. S1 to S46
Tables S1 to S5
SI References

Supporting Information Text

Datasets

For this study, datasets with long temporal and global coverage, moderate spatial resolution (down to 250m), and temporal resolution (up to hourly) were selected. Vegetation distribution, coverage, and density were analyzed using various LAI and NDVI products, while information on GPP (the gross uptake flux of CO_2 via terrestrial photosynthesis) was obtained from the newest machine learning-based upscaling products. Specifically, we used the LAI products GIMMS LAI4g (1), GLASS LAI V6 (2) and GLOBMAP LAI (3); three NDVI products: MODIS NDVI (4, 5), MODIS kNDVI (4, 6) and GIMMS NDVI4g (7); and GPP estimates from GOSIF V2 (8) and FLUXCOM-X-BASE (9). These datasets provided a comprehensive basis for understanding vegetation dynamics and productivity at a global scale. Details of these datasets are also available in supplementary Table S1.

GIMMS LAI4g (1) is a half-monthly global LAI dataset covering 1982–2020 with a spatial resolution of 1/12°, designed to address limitations in spatiotemporal consistency of current LAI products. It was created using an artificial neural network and a data consolidation method, integrating the latest PKU GIMMS NDVI product and 3.6 million high-quality global Landsat LAI samples. This approach mitigates the impacts of satellite orbital drift and sensor degradation, ensuring a more reliable and consistent dataset. The GIMMS LAI4g dataset supports biome-specific modeling and intercomparison with other products like GLASS and GLOBMAP.

The GLASS LAI V6 (2) dataset was created using general regression neural networks (GRNNs) trained on integrated MODIS and AVHRR surface reflectance data. It features long-term temporal coverage spanning 2000-2021, high spatial resolution of 250m, and 8-day temporal resolution, ensuring spatial continuity, quality, and accuracy validated through intercomparisons (10).

The GLOBMAP LAI (3) product (1981–2023) is a long-term global LAI dataset generated by fusing MODIS and historical AVHRR data using the GLOBCARBON algorithm (11). It features strong consistency between LAIs from both sensors and reduced temporal noise compared to direct GLOBCARBON retrievals. GLOBMAP LAI provides data at 8 km spatial- and 8-day temporal resolutions, covering 180°W–180°E and 63°S–90°N.

The GIMMS NDVI4g (7) is a global NDVI product developed using a machine learning model that integrates high-quality Landsat NDVI samples and AVHRR-MODIS data to overcome limitations of previous GIMMS NDVI3g, such as orbital drift and sensor degradation. The GIMMS NDVI4g eliminates major issues in tropical regions, ensuring consistency in vegetation trend analysis and improving reliability for global change studies. The dataset provides a spatial resolution of 1/12° with a half-month temporal resolution covering the period from 1982 to 2022.

MODIS NDVI and kNDVI were derived from the MCD43C4 dataset(4), a global Nadir BRDF-Adjusted Reflectance (NBAR) product with a spatial resolution of 0.05° and an 8-day temporal resolution, spanning from 2000 to the present (we employed the MODIS product in this study, which is 2000-2023). It is produced using 16-day Terra and Aqua MODIS data composites, with reflectances adjusted to a nadir view at local solar noon, and temporally weighted to an 8-day temporal resolution. The product includes NBAR for MODIS bands 1–7 along with ancillary layers for quality, solar noon, snow cover, uncertainty, and percent finer resolution inputs (4).

Two GPP products were employed in this study, one being the GOSIF V2 GPP dataset (8), which estimates GPP using data from OCO-2, MODIS, and reanalysis sources. This dataset, with a spatial resolution of 0.05° and an 8-day temporal resolution, spans 2000–2023, providing comprehensive insights into terrestrial carbon dynamics and ecosystem productivity.

The FLUXCOM-X-BASE products(9), developed under the FLUXCOM-X framework, offer global estimates of GPP with a spatial resolution of 0.05° and an hourly temporal resolution for 2001–2020. These products demonstrate enhanced consistency with atmospheric carbon cycle constraints, enabling more accurate assessments of carbon fluxes and ecosystem productivity at fine temporal scales.

All data were preprocessed considering the respective data quality flags, such as removing snow-covered or cloud-contaminated observations. While most products were already curated and gap-filled by data providers, MODIS products were preprocessed using raw data from the original daily NBAR product. NDVI and kNDVI were computed from the daily data and aggregated to an 8-day temporal resolution using the median of each period. The remaining gaps after resampling were filled using a two-step approach: first, missing winter values were replaced with random samples from the lowest 0.05 quantile; second, the remaining gaps were linearly interpolated. As can be seen from fig. S1, the effects on the global green wave trajectory should be minor as the gap fraction is very low.

All resulting data were stored as the aforementioned data cubes $\rho_{\lambda_i,\phi_i,t}$ (12, 13). Data cubes are multidimensional data structures that integrate heterogeneous Earth system data into a consistent framework with labeled dimensions (e.g., space, time, and variables), coordinate-defined grids, data values, and metadata attributes. These products were stored using the Zarr format for efficient access and parallel computation.

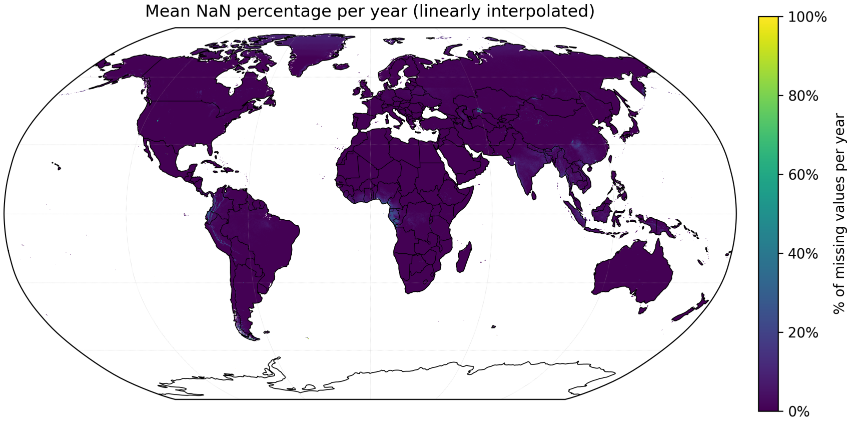

Fig. S1. Fraction of gaps we had to gapfill in MODIS products before computing the green wave trajectory.

CMIP6 data, model selection, and model averaging

The variable LAI was taken from the CMIP6 multi-model archive (14) of Earth system model simulations using the historical simulations (up until 2014) and subsequent future projections under different SSP scenarios. All model data is regridded to a regular 2.5°×2.5° longitude-latitude grid, following the procedures of the CMIP6-ETH next-generation archive (15).

Here, we clarify the criteria used to select CMIP6 models for the projection analysis. The selection was not based on agreement with the historical green-wave indicator. Instead, we applied a two-step strategy: (i) technical and data-availability requirements, and (ii) a process-based signal-to-noise criterion ensuring that models exhibited a detectable green-wave signal.

Filter 1: Technical requirements. From the ETH Zurich CMIP6 archive, we: (i) selected all model outputs containing monthly LAI on their native grids; (ii) retained only models providing ensembles for the scenarios *historical*, *ssp126*, *ssp245*, *ssp370*, and *ssp585*; (iii) selected ensemble members with `init = 1`, `physics = 1`, `forcing = 1`; (iv) removed ensemble members without LAI data; and (v) removed models lacking complete scenario coverage.

Filter 2: Detectability of the green-wave signal. We computed the green wave centroid trajectory for each model and ensemble member. The question was which models to include in an ensemble mean. Our idea was to keep the analysis fully independent from observations. This is why we did not select models based on agreement with Earth-observation products. Instead, we required that a model show a detectable green-wave signal in its own dynamics. We applied a simple signal-to-noise ratio (SNR) criterion to the trend in the boreal viridistice position, $z(v_B)$.

For each model we evaluated:

$$\mathrm{SNR} = \frac{a(z(v_B))\ L}{\mathrm{SD}(z(v_B))} > 1,$$

where $a(z(v_B))$ is the temporal slope (Sen's slope) of the trend in the boreal-summer vertical displacement of the green-wave trajectory, $L = 30$ years is the climate reference period used (the first 30 years of the projection), and $\mathrm{SD}(z(v_B))$ is the standard deviation of the detrended series, representing interannual variability. Models with $\mathrm{SNR} \leq 1$ were considered not to exhibit a physically interpretable signal for the process under study. Because SNR depends on the scenario, we evaluated it only for SSP1–2.6, where the trend is expected to be most difficult to detect.

The resulting SNR values were:

Table S1. Signal-to-noise ratio (SNR) of the boreal viridistice trajectory trend for CMIP6 models. Models with $\mathrm{SNR} > 1$ (based on SSP1–2.6) were retained, ensuring a detectable green-wave signal independent of observations.

CMIP6 Model	Land-model	SNR
ACCESS-ESM1-5	CABLE v2.4	8.6
CanESM5	CLASS v3.6+CTEM	1.9
CESM2	CLM5	1.2
CMCC-CM2-SR5	CLM4.5	0.8
CMCC-ESM2	CLM4.5	0.8
FGOALS-g3	VEGAS	5.7
INM-CM4-8	INMCM carbon cycle module	0.9
IPSL-CM6A-LR	ORCHIDEE	5.4
MPI-ESM1-2-LR	JSBACH	1.8

Models lacking a detectable green-wave signal under low forcing are unlikely to provide a physically interpretable response under higher forcing, although scenario-specific variability may influence the SNR. The resulting subset of five models, therefore, reflects process relevance rather than observational tuning. Three CMIP6 models that passed the initial checks simply do not

encode the green wave and merit scrutiny in follow-up work. This SNR analysis also shows that the green-wave trajectory can serve as an independent diagnostic for Earth system model evaluation.

Model averaging: For constructing the mean ensemble of the retained models, we applied a bootstrap approach to compute the model-ensemble mean trajectories and associated uncertainties shown in Fig. 4. For each bootstrap sample, we randomly selected one green-wave trajectory from one ensemble member of each model, ensuring equal representation of all models in every average. Because some models have fewer ensemble members in the CMIP6 archive, their available members were sampled more frequently to maintain model balance.

Interactive website

Didactic exploration of this paper's key concepts through an interactive website. An interactive website that explains the contents of this paper in a didactic manner is available and will be accessible without password protection upon publication.

- http://www.greenwave.earth

The website features a visual narrative that guides readers step-by-step through the concepts of the green wave, centroid trajectories, and the northeast shift. Users can interact with a rotating 3D globe and change the view or data layers. Additional sections allow exploration of the centroid position through time ("3D Centroid Explorer"), model projections under four different SSP scenarios ("Futures Explorer"), and an interactive explanation of global greening for context.

Expert exploration of this paper's key concepts through interactive visualization. An interactive version of Figure 1 is also available to enable scientific specialists to explore the visualization at different spatial resolutions and to switch between data products.

- https://trajectory.greenwave.earth

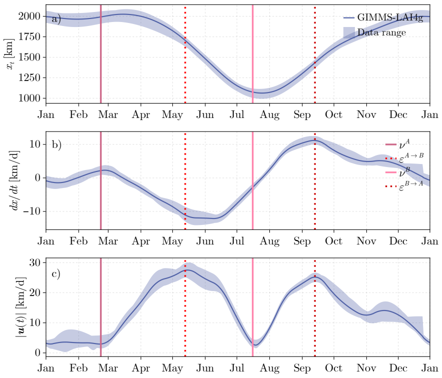

Fig. S2. MSC for the x-component of GIMMS LAI4g

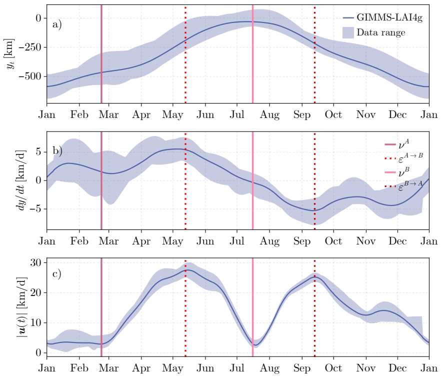

Fig. S3. MSC for the y-component of GIMMS LAI4g

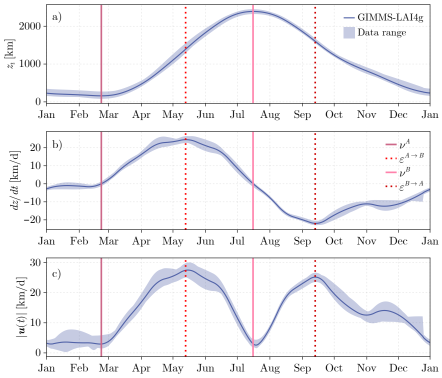

Fig. S4. MSC for the z-component of GIMMS LAI4g

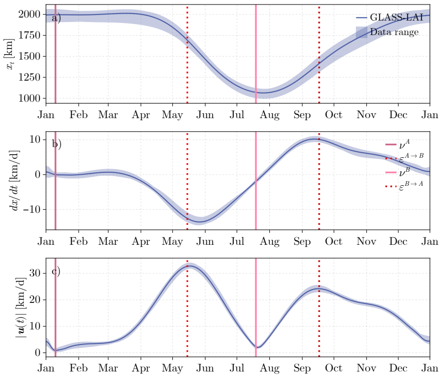

Fig. S5. MSC for the x-component of GLASS LAI V6

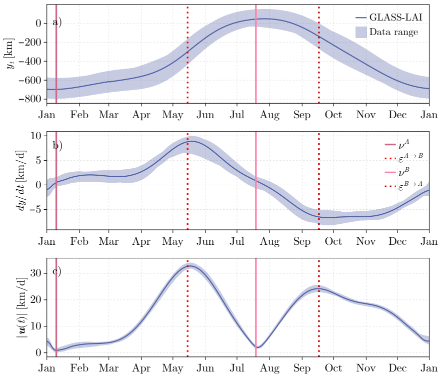

Fig. S6. MSC for the y-component of GLASS LAI V6

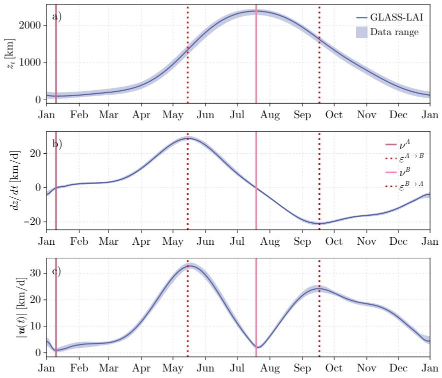

Fig. S7. MSC for the z-component of GLASS LAI V6

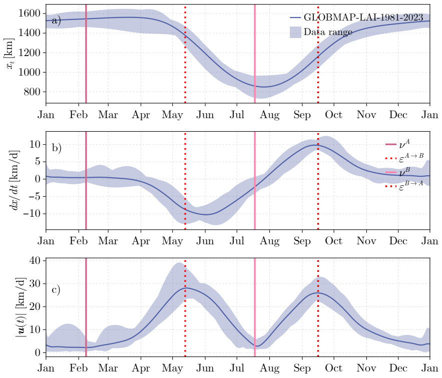

Fig. S8. MSC for the x-component of GLOBMAP LAI

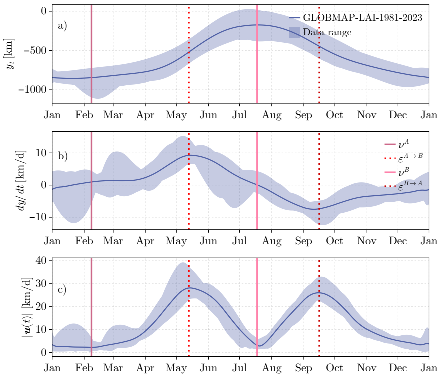

Fig. S9. MSC for the y-component of GLOBMAP LAI

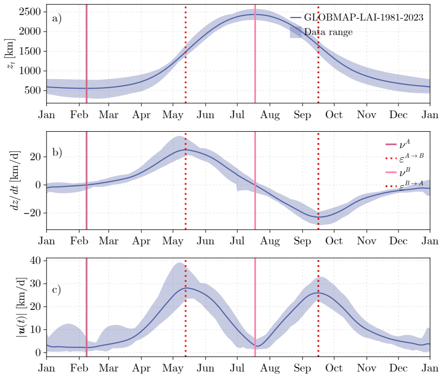

Fig. S10. MSC for the z-component of GLOBMAP LAI

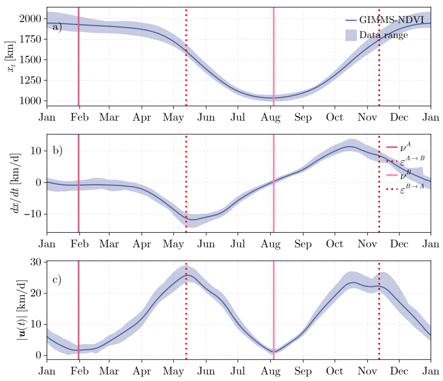

Fig. S11. MSC for the x-component of GIMMS NDVI4g

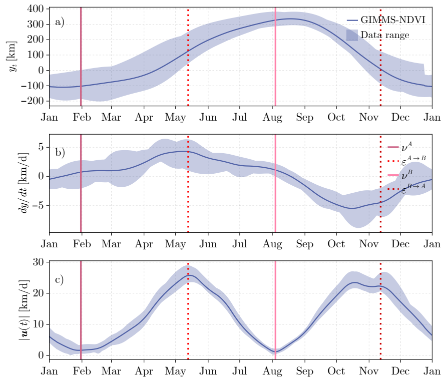

Fig. S12. MSC for the y-component of GIMMS NDVI4g

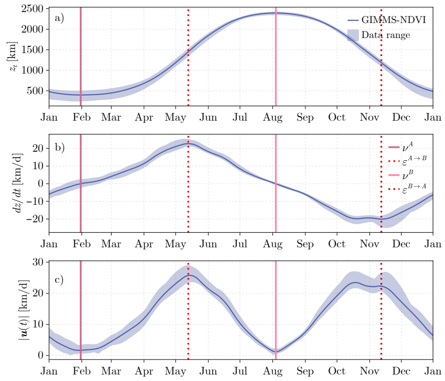

Fig. S13. MSC for the z-component of GIMMS NDVI4g

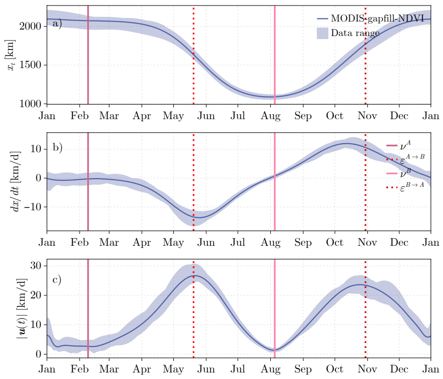

Fig. S14. MSC for the x-component of MODIS NDVI

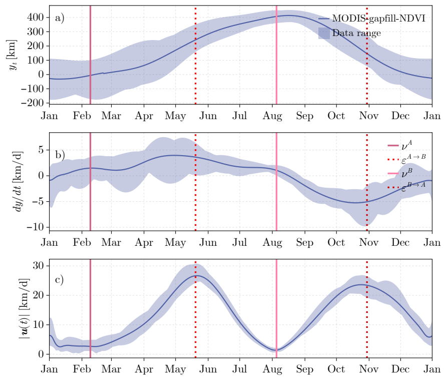

Fig. S15. MSC for the y-component of MODIS NDVI

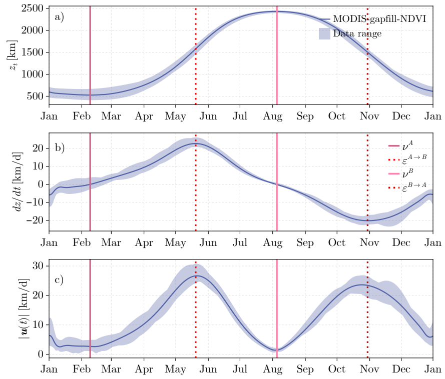

Fig. S16. MSC for the z-component of MODIS NDVI

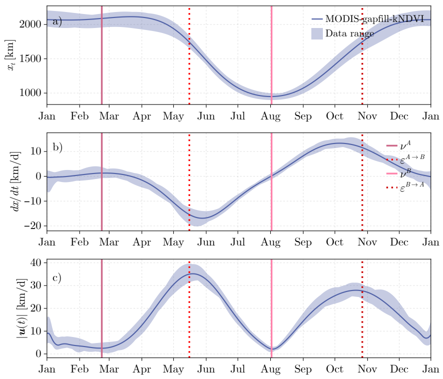

Fig. S17. MSC for the x-component of MODIS kNDVI

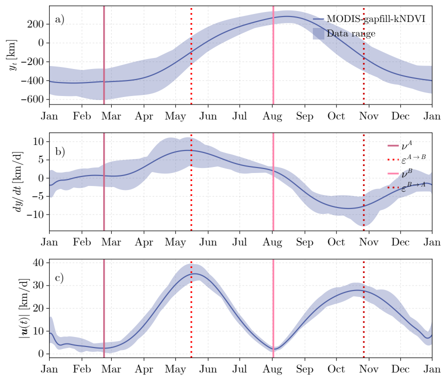

Fig. S18. MSC for the y-component of MODIS kNDVI

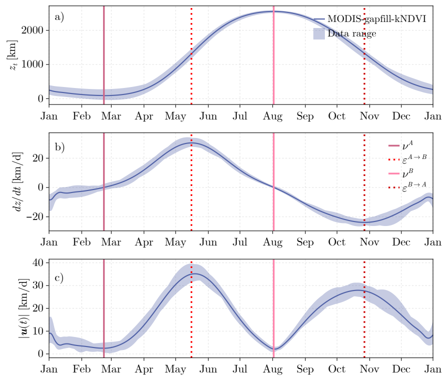

Fig. S19. MSC for the z-component of MODIS kNDVI

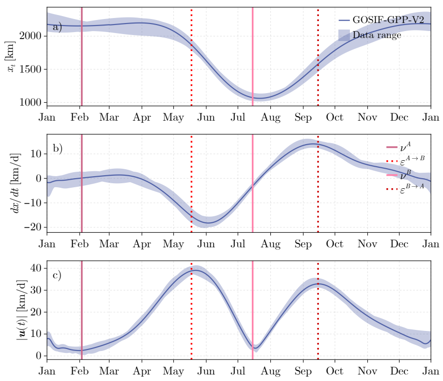

Fig. S20. MSC for the x-component of GOSIF GPP V2

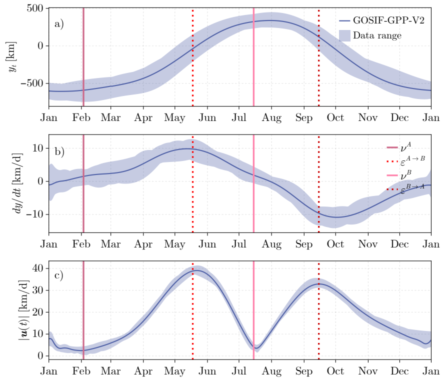

Fig. S21. MSC for the y-component of GOSIF GPP V2

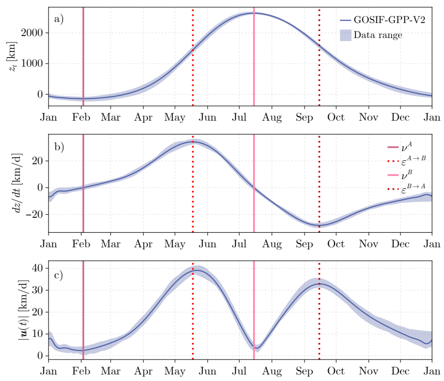

Fig. S22. MSC for the z-component of GOSIF GPP V2

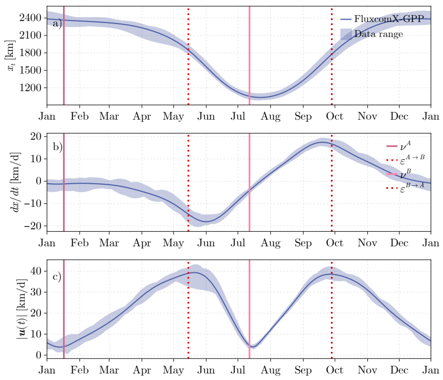

Fig. S23. MSC for the x-component of FLUXCOM-X-BASE GPP

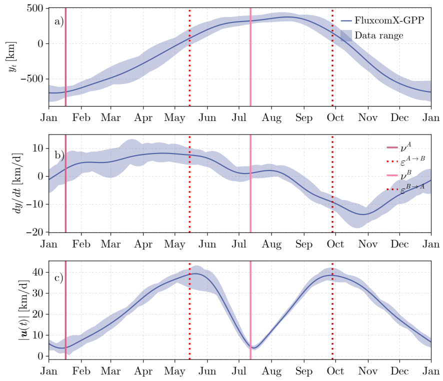

Fig. S24. MSC for the y-component of FLUXCOM-X-BASE GPP

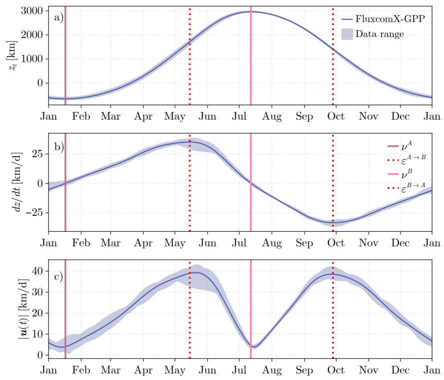

Fig. S25. MSC for the z-component of FLUXCOM-X-BASE GPP

Table S2. Estimates of viridistice and equiviridis across global datasets. Values represent circular means (with circular standard deviations in days) derived from leaf area index (LAI), greenness indicators (NDVI, kNDVI), and gross primary production (GPP).

Variable	Data Product	ν^B	ν^A	$\varepsilon^{A\to B}$	$\varepsilon^{B\to A}$	Duration
**LAI**						
	GIMMS LAI4g	July 16 ± 1.44	Feb 21 ± 6.78	May 11 ± 7.32	Sep 14 ± 1.30	1982–2020
	GLASS LAI V6	July 19 ± 0.97	Jan 9 ± 4.42	May 15 ± 1.37	Sep 17 ± 1.07	2000–2021
	GLOBMAP LAI	July 17 ± 5.68	Jan 31 ± 18.06	May 16 ± 18.65	Sep 15 ± 5.96	1981–2023
**NDVI**						
	GIMMS NDVI4g	Aug 4 ± 1.37	Feb 1 ± 7.62	May 13 ± 1.42	Nov 5 ± 12.48	1982–2022
	MODIS NDVI	Aug 5 ± 1.73	Feb 7 ± 12.39	May 20 ± 3.94	Oct 30 ± 10.34	2000–2023
	MODIS kNDVI	Aug 2 ± 1.54	Feb 20 ± 11.43	May 16 ± 3.64	Oct 28 ± 10.08	2000–2023
**GPP**						
	GOSIF V2	July 15 ± 1.66	Feb 2 ± 11.05	May 18 ± 2.91	Sep 15 ± 1.85	2000–2023
	FLUXCOM-X (BASE)	July 12 ± 1.40	Jan 16 ± 4.68	May 11 ± 9.02	Sep 28 ± 5.85	2001–2021

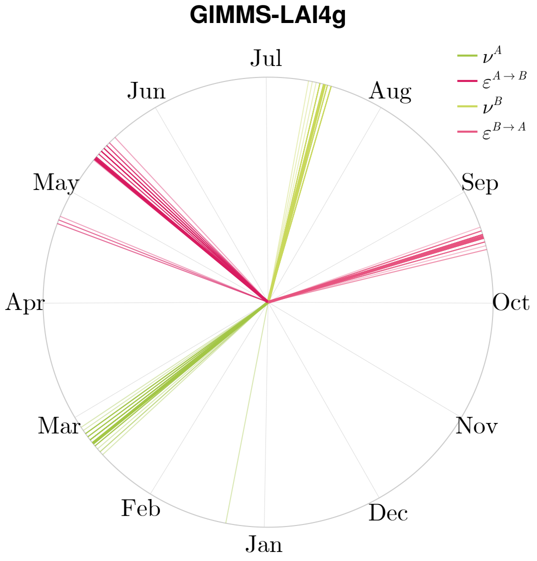

Fig. S26. Circular representation of the multiyear boreal and austral viridistice (ν^B_{year} and ν^A_{year}) respectively, as well as the corresponding moments of equiviridis ($\varepsilon^{B\to A}_{\text{year}}$ and $\varepsilon^{A\to B}_{\text{year}}$) for GIMMS LAI4g.

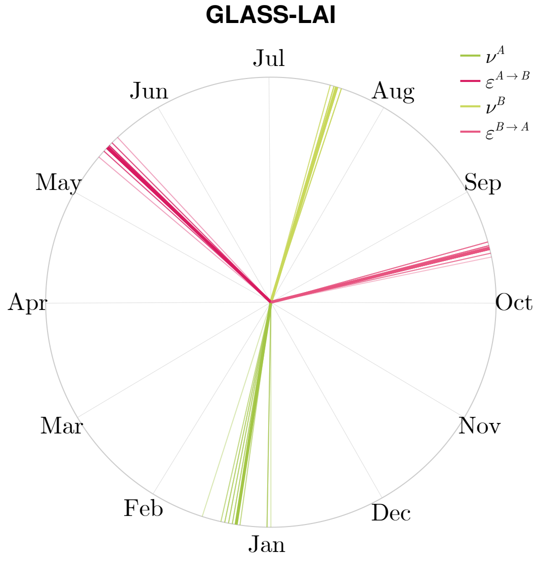

Fig. S27. Circular representation of the multiyear boreal and austral viridistice (ν^B_{year} and ν^A_{year}) respectively, as well as the corresponding moments of equiviridis ($\varepsilon^{B\to A}_{\text{year}}$ and $\varepsilon^{A\to B}_{\text{year}}$) for GLASS LAI V6

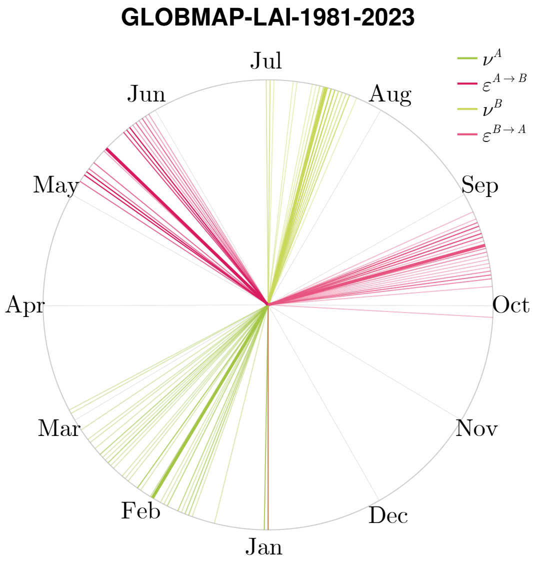

Fig. S28. Circular representation of the multiyear boreal and austral viridistice (ν^B_{year} and ν^A_{year}) respectively, as well as the corresponding moments of equiviridis ($\varepsilon^{B\to A}_{\text{year}}$ and $\varepsilon^{A\to B}_{\text{year}}$) for GLOBMAP.

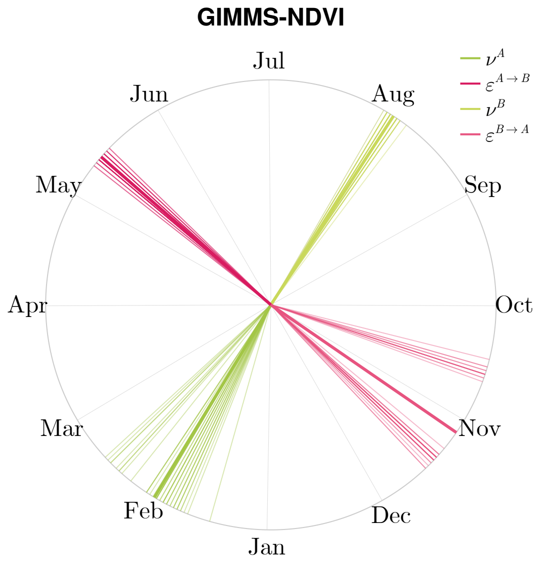

Fig. S29. Circular representation of the multiyear boreal and austral viridistice (ν^B_{year} and ν^A_{year}) respectively, as well as the corresponding moments of equiviridis ($\varepsilon^{B\to A}_{\text{year}}$ and $\varepsilon^{A\to B}_{\text{year}}$) for GIMMS NDVI4g.

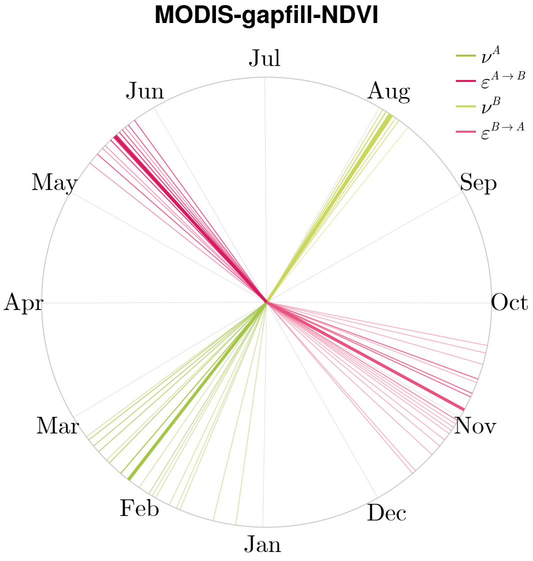

Fig. S30. Circular representation of the multiyear boreal and austral viridistice (ν^B_{year} and ν^A_{year}) respectively, as well as the corresponding moments of equiviridis ($\varepsilon^{B\to A}_{\text{year}}$ and $\varepsilon^{A\to B}_{\text{year}}$) for MODIS NDVI.

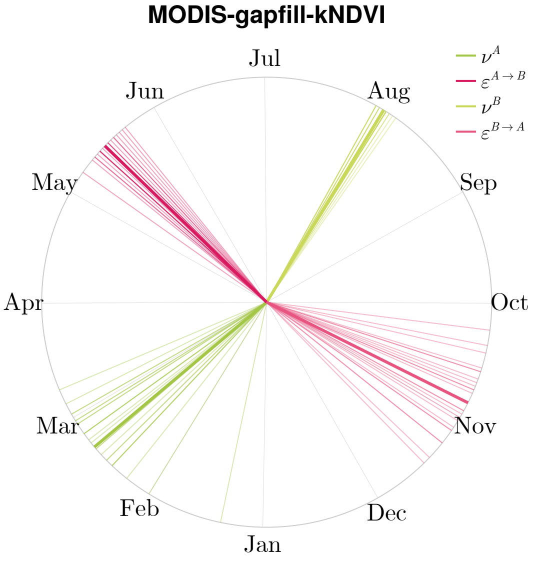

Fig. S31. Circular representation of the multiyear boreal and austral viridistice (ν^B_{year} and ν^A_{year}) respectively, as well as the corresponding moments of equiviridis ($\varepsilon^{B\to A}_{\text{year}}$ and $\varepsilon^{A\to B}_{\text{year}}$) for MODIS kNDVI.

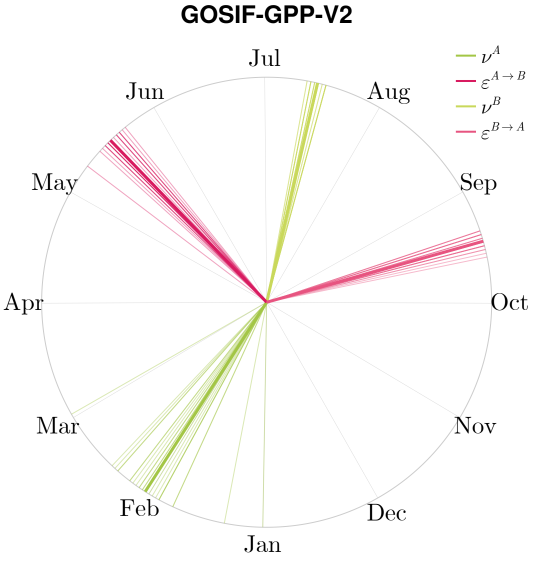

Fig. S32. Circular representation of the multiyear boreal and austral viridistice (ν^B_{year} and ν^A_{year}) respectively, as well as the corresponding moments of equiviridis ($\varepsilon^{B\to A}_{\text{year}}$ and $\varepsilon^{A\to B}_{\text{year}}$) for GOSIF V8 GPP.

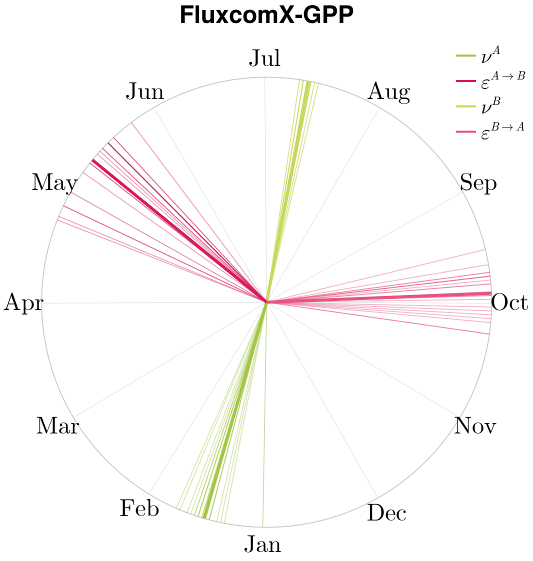

Fig. S33. Circular representation of the multiyear boreal and austral viridistice (ν^B_{year} and ν^A_{year}) respectively, as well as the corresponding moments of equiviridis ($\varepsilon^{B\to A}_{\text{year}}$ and $\varepsilon^{A\to B}_{\text{year}}$) for FLUXCOM X Base GPP.

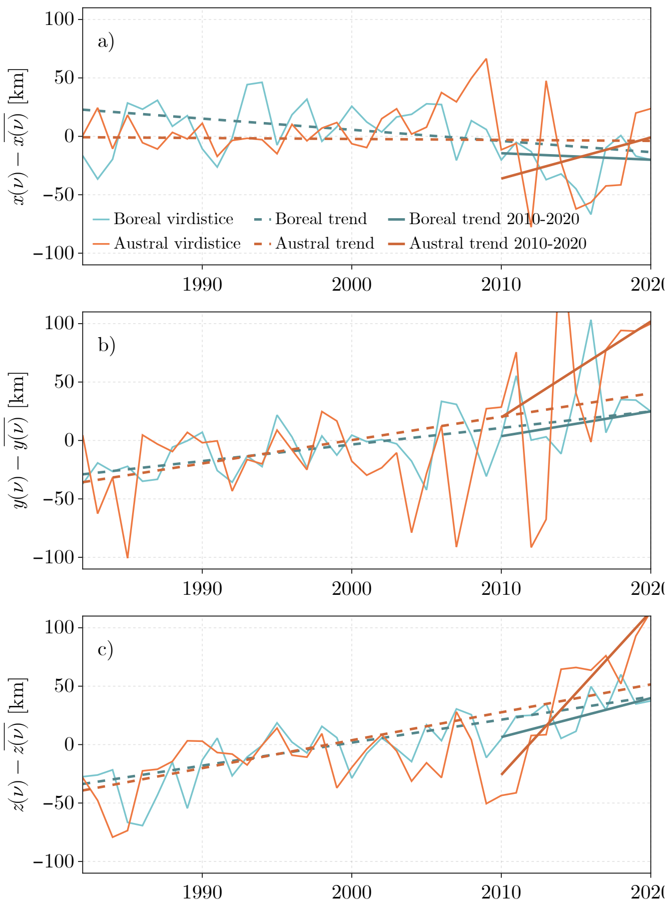

Fig. S34. Shifts in the 3D positioning of the boreal and austral viridistice. Temporal development of the trajectory positions during viridistice from GIMMS LAI4g ($x(\nu)$, $y(\nu)$, $z(\nu)$), centered to zero mean for scale comparison. Decadal trend lines are computed using Sen's slope.

Table S3. Mann–Kendall tau, Sen's slope and p-values for the trends in viridistices for the 2010–2020 period.

Data product	Quantity	τ	Slope [km yr^{-1}]	p-value
GIMMS-LAI4g	$x(\nu^B)$	-0.018	-0.56	1
GLASS-LAI	$x(\nu^B)$	-0.061	-0.24	0.837
GLOBMAP-LAI-1981-2023	$x(\nu^B)$	-0.451	-4.55	0.0285
GIMMS-NDVI	$x(\nu^B)$	0.154	1.32	0.502
MODIS-gapfill-NDVI	$x(\nu^B)$	0.055	0.35	0.827
MODIS-gapfill-kNDVI	$x(\nu^B)$	0.033	0.26	0.913
FluxcomX-GPP	$x(\nu^B)$	-0.242	-3.35	0.304
GOSIF-GPP-V2	$x(\nu^B)$	-0.033	-0.18	0.913
GIMMS-LAI4g	$x(\nu^A)$	0.164	3.52	0.533
GLASS-LAI	$x(\nu^A)$	-0.182	-3.15	0.451
GLOBMAP-LAI-1981-2023	$x(\nu^A)$	0.077	1.62	0.743
GIMMS-NDVI	$x(\nu^A)$	0.000	-0.20	0.951
MODIS-gapfill-NDVI	$x(\nu^A)$	-0.033	-0.34	0.913
MODIS-gapfill-kNDVI	$x(\nu^A)$	0.055	0.31	0.827
FluxcomX-GPP	$x(\nu^A)$	0.152	3.49	0.537
GOSIF-GPP-V2	$x(\nu^A)$	0.033	1.08	0.913

Table S4. Mann–Kendall tau, Sen's slope and p-values for the trends in viridistices for the 2010–2020 period.

Data product	Quantity	τ	Slope [km yr^{-1}]	p-value
GIMMS-LAI4g	$y(\nu^B)$	0.127	2.12	0.64
GLASS-LAI	$y(\nu^B)$	0.606	8.34	0.00749
GLOBMAP-LAI-1981-2023	$y(\nu^B)$	0.341	7.11	0.101
GIMMS-NDVI	$y(\nu^B)$	0.179	1.85	0.428
MODIS-gapfill-NDVI	$y(\nu^B)$	0.275	2.54	0.189
MODIS-gapfill-kNDVI	$y(\nu^B)$	0.407	4.40	0.0487
FluxcomX-GPP	$y(\nu^B)$	0.273	3.74	0.244
GOSIF-GPP-V2	$y(\nu^B)$	0.495	7.46	0.016
GIMMS-LAI4g	$y(\nu^A)$	0.455	8.20	0.0617
GLASS-LAI	$y(\nu^A)$	0.515	9.56	0.0236
GLOBMAP-LAI-1981-2023	$y(\nu^A)$	0.187	3.00	0.381
GIMMS-NDVI	$y(\nu^A)$	0.436	4.94	0.0441
MODIS-gapfill-NDVI	$y(\nu^A)$	0.451	7.37	0.0285
MODIS-gapfill-kNDVI	$y(\nu^A)$	0.560	9.48	0.0062
FluxcomX-GPP	$y(\nu^A)$	-0.061	-0.98	0.837
GOSIF-GPP-V2	$y(\nu^A)$	0.209	5.28	0.324

Table S5. Mann–Kendall tau, Sen's slope and p-values for the trends in viridistices for the 2010–2020 period.

Data product	Quantity	τ	Slope [km yr^{-1}]	p-value
GIMMS-LAI4g	$z(\nu^B)$	0.527	3.33	0.0293
GLASS-LAI	$z(\nu^B)$	0.576	4.47	0.0112
GLOBMAP-LAI-1981-2023	$z(\nu^B)$	0.341	6.44	0.101
GIMMS-NDVI	$z(\nu^B)$	0.385	1.75	0.0769
MODIS-gapfill-NDVI	$z(\nu^B)$	0.451	1.60	0.0285
MODIS-gapfill-kNDVI	$z(\nu^B)$	0.495	2.53	0.016
FluxcomX-GPP	$z(\nu^B)$	0.121	1.00	0.631
GOSIF-GPP-V2	$z(\nu^B)$	0.231	1.56	0.274
GIMMS-LAI4g	$z(\nu^A)$	0.782	13.97	0.00108
GLASS-LAI	$z(\nu^A)$	0.758	10.02	0.000779
GLOBMAP-LAI-1981-2023	$z(\nu^A)$	0.341	7.56	0.101
GIMMS-NDVI	$z(\nu^A)$	0.641	15.88	0.00279
MODIS-gapfill-NDVI	$z(\nu^A)$	0.538	13.05	0.0086
MODIS-gapfill-kNDVI	$z(\nu^A)$	0.451	15.52	0.0285
FluxcomX-GPP	$z(\nu^A)$	0.515	9.38	0.0236
GOSIF-GPP-V2	$z(\nu^A)$	0.473	5.73	0.0215

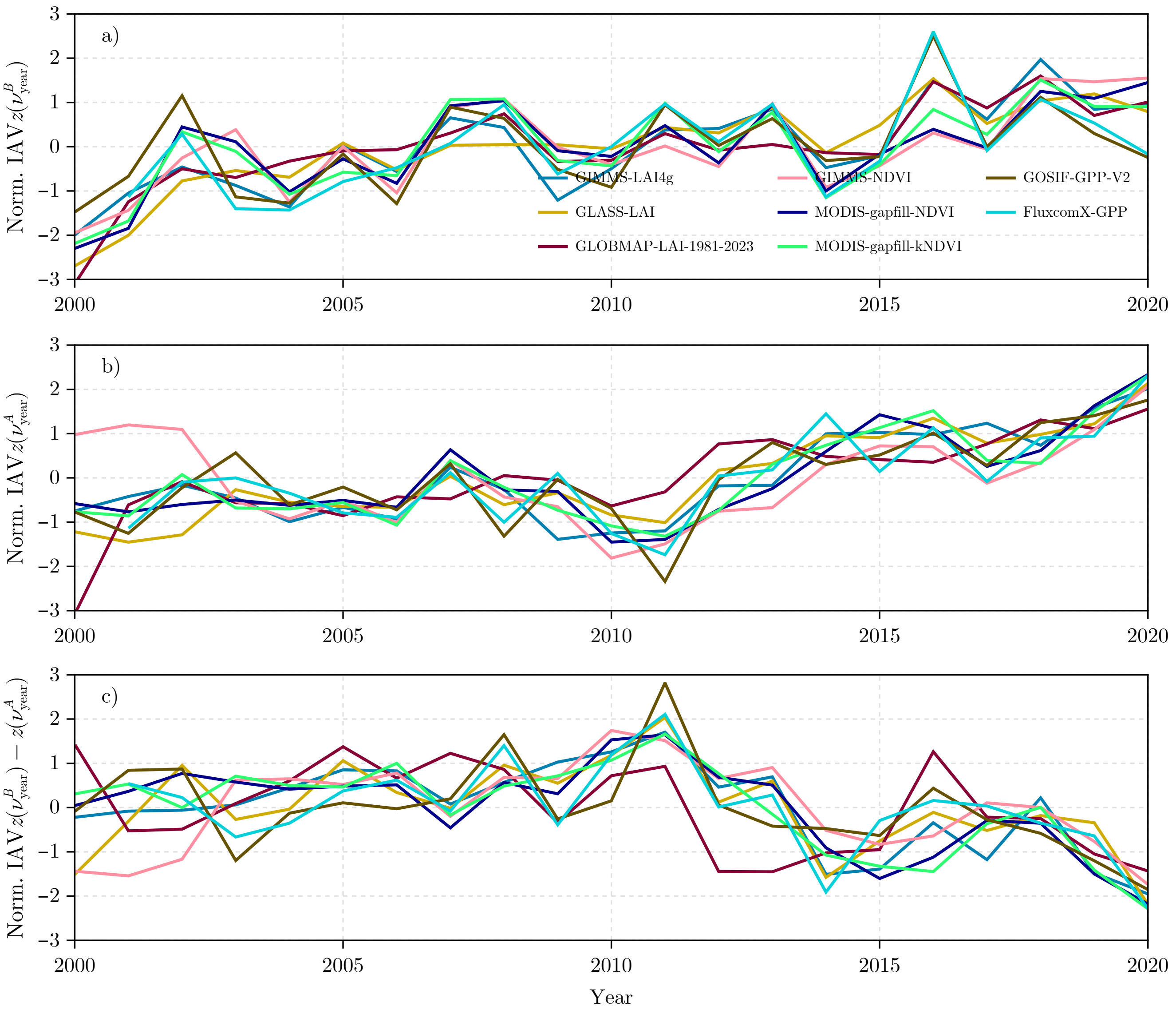

Fig. S35. Interannual variability in viridistice across data products. For each dataset, we derive the boreal and austral viridistice times (ν^B_{year}, ν^A_{year}) and compute their associated trajectory heights relative to the equatorial plane ($z(\nu^B_{\text{year}})$, $z(\nu^A_{\text{year}})$), as well as their contrast $z(\nu^B_{\text{year}}) - z(\nu^A_{\text{year}})$. Shown are these time series, each normalized to zero mean and unit variance over 2000–2020.

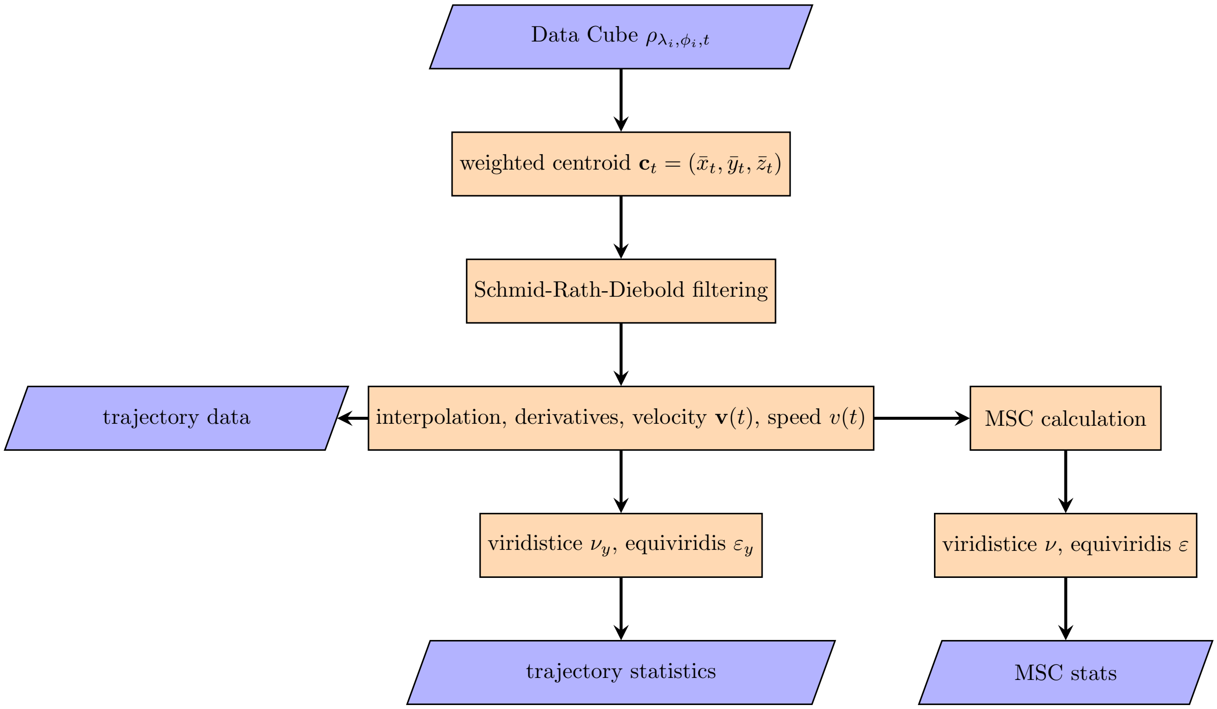

Fig. S36. Workflow for processing data from the initial data cubes to the trajectories and their statistics.

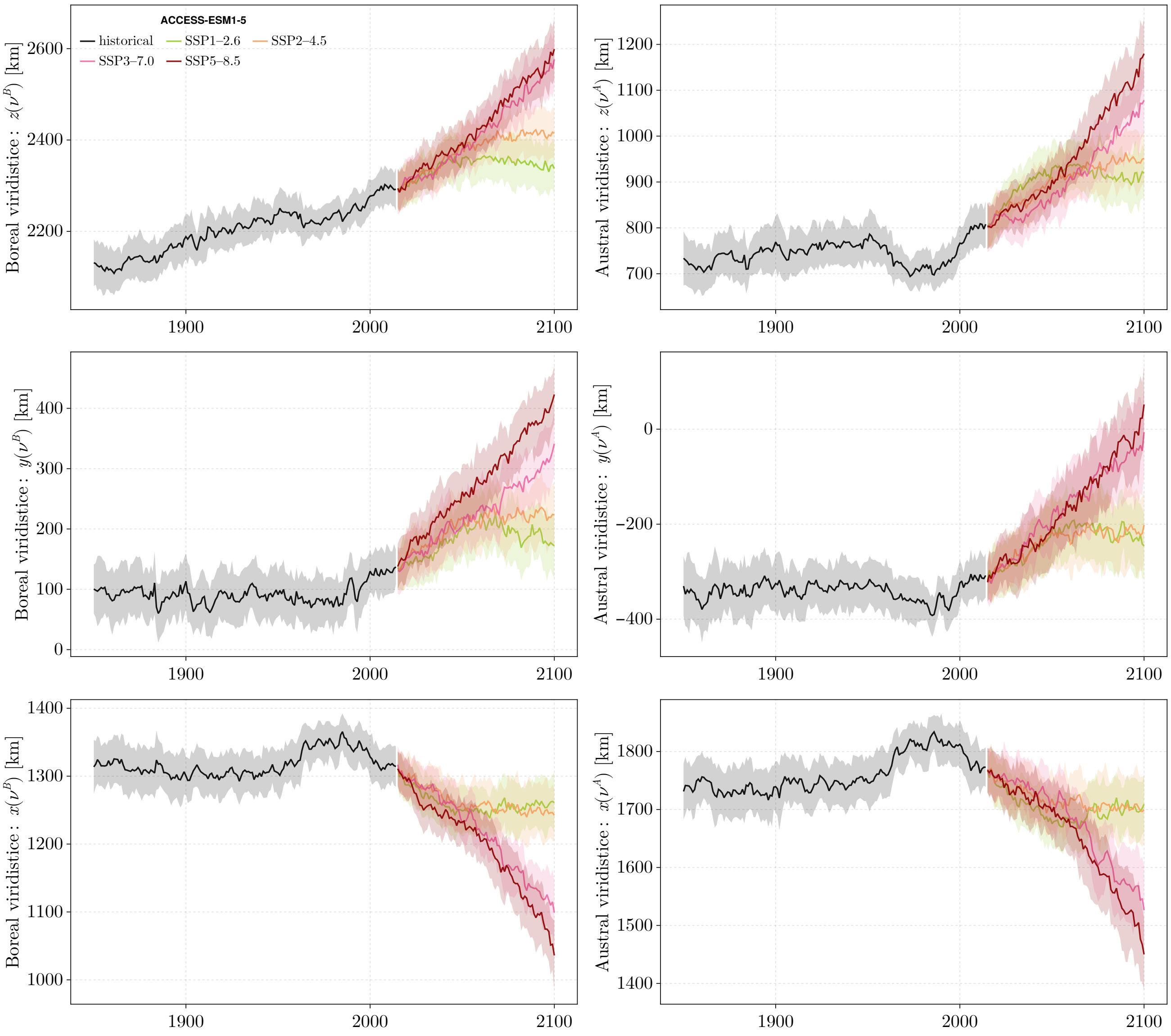

Fig. S37. z, y, and x-coordinates (top to bottom) of the green wave centroid trajectory positions during boreal and austral (left to right) viridistices ($z(\nu^A_{\text{year}})$ and $z(\nu^B_{\text{year}})$) in historical simulations and four Shared Socioeconomic Pathways (SSP) scenarios of the CMIP6 run by the ACCESS-ESM1-5 model. If there are several model runs available, the mean ± 1SD is shown.

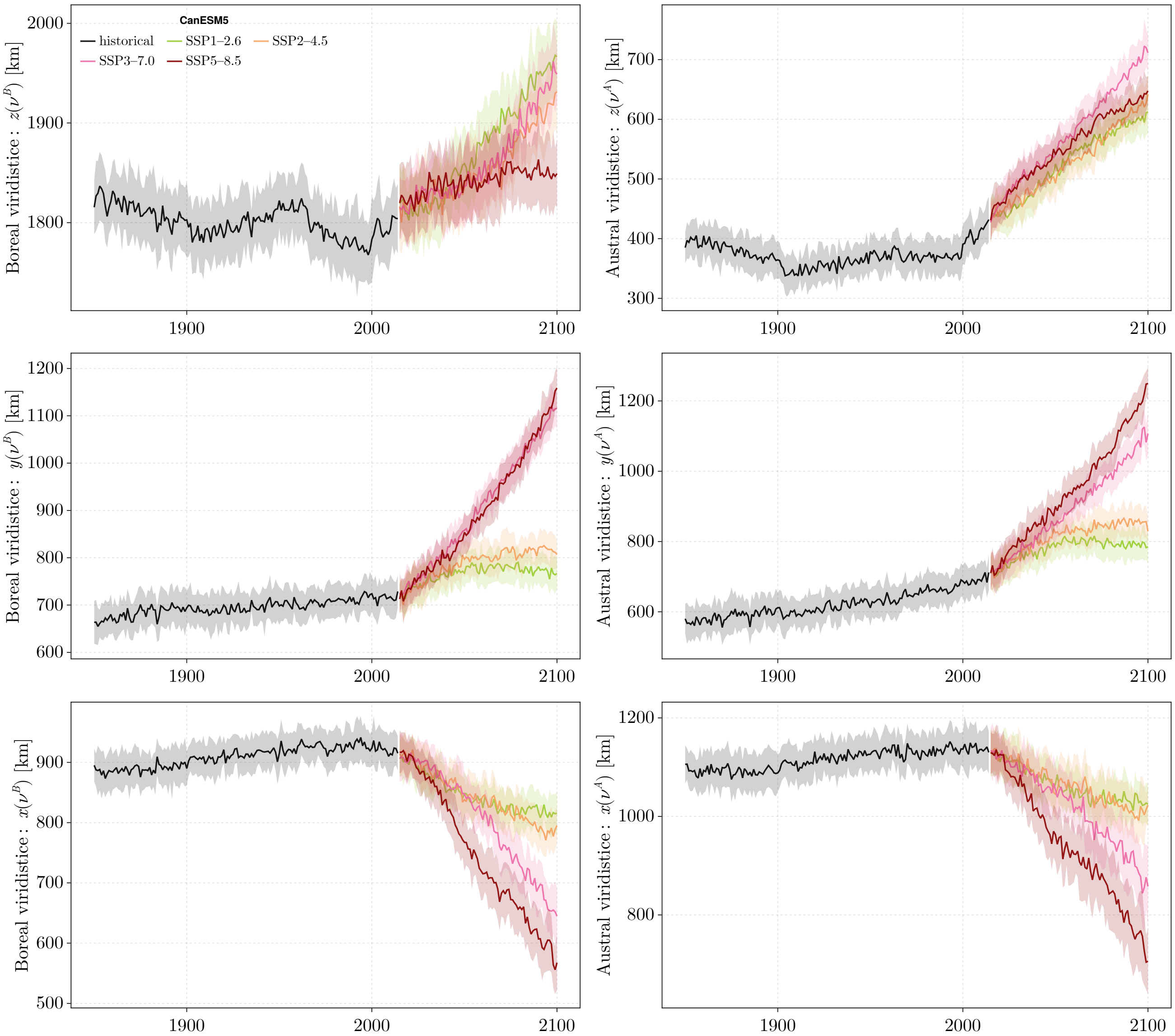

Fig. S38. z, y, and x-coordinates (top to bottom) of the green wave centroid trajectory positions during boreal and austral (left to right) viridistices ($z(\nu^A_{\text{year}})$ and $z(\nu^B_{\text{year}})$) in historical simulations and four Shared Socioeconomic Pathways (SSP) scenarios of the CMIP6 run by the CanESM5 model. If there are several model runs available, the mean ± 1SD is shown.

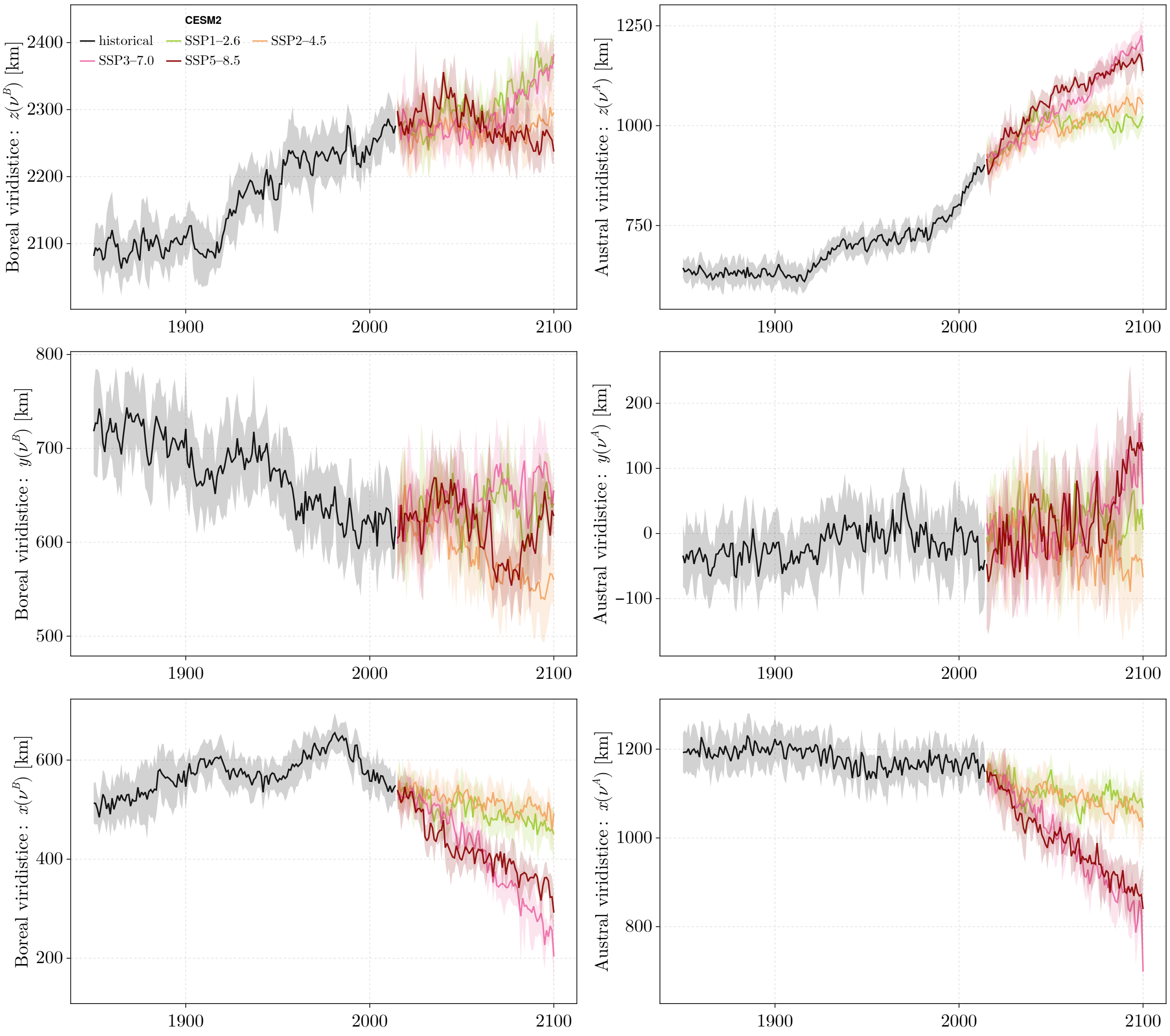

Fig. S39. z, y, and x-coordinates (top to bottom) of the green wave centroid trajectory positions during boreal and austral (left to right) viridistices ($z(\nu^A_{\text{year}})$ and $z(\nu^B_{\text{year}})$) in historical simulations and four Shared Socioeconomic Pathways (SSP) scenarios of the CMIP6 run by the CESM2 model. If there are several model runs available, the mean ±1SD is shown.

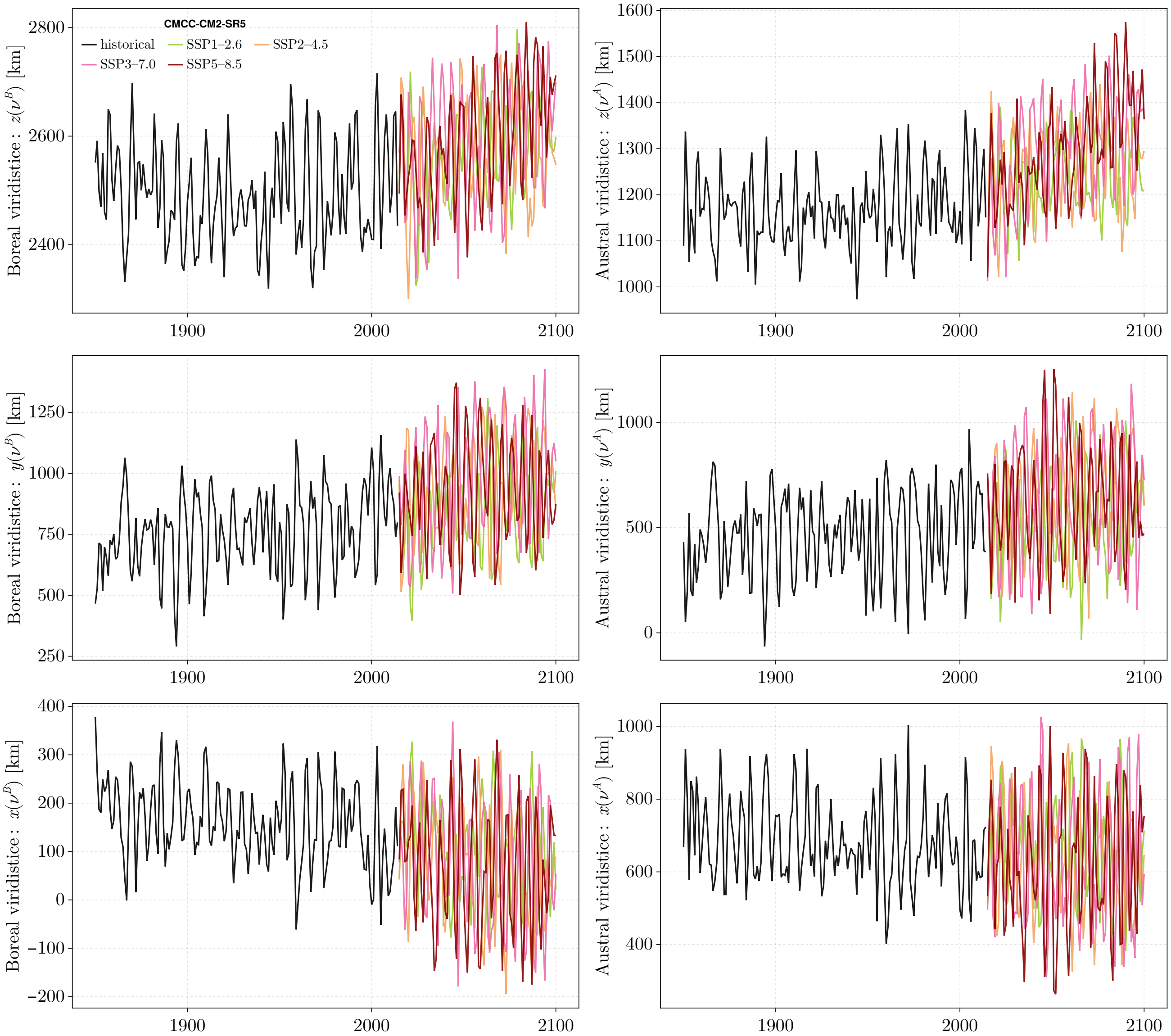

Fig. S40. z, y, and x-coordinates (top to bottom) of the green wave centroid trajectory positions during boreal and austral (left to right) viridistices ($z(\nu^A_{\text{year}})$ and $z(\nu^B_{\text{year}})$) in historical simulations and four Shared Socioeconomic Pathways (SSP) scenarios of the CMIP6 run by the CMCC-CM2-SR5 model. If there are several model runs available, the mean ±1SD is shown.

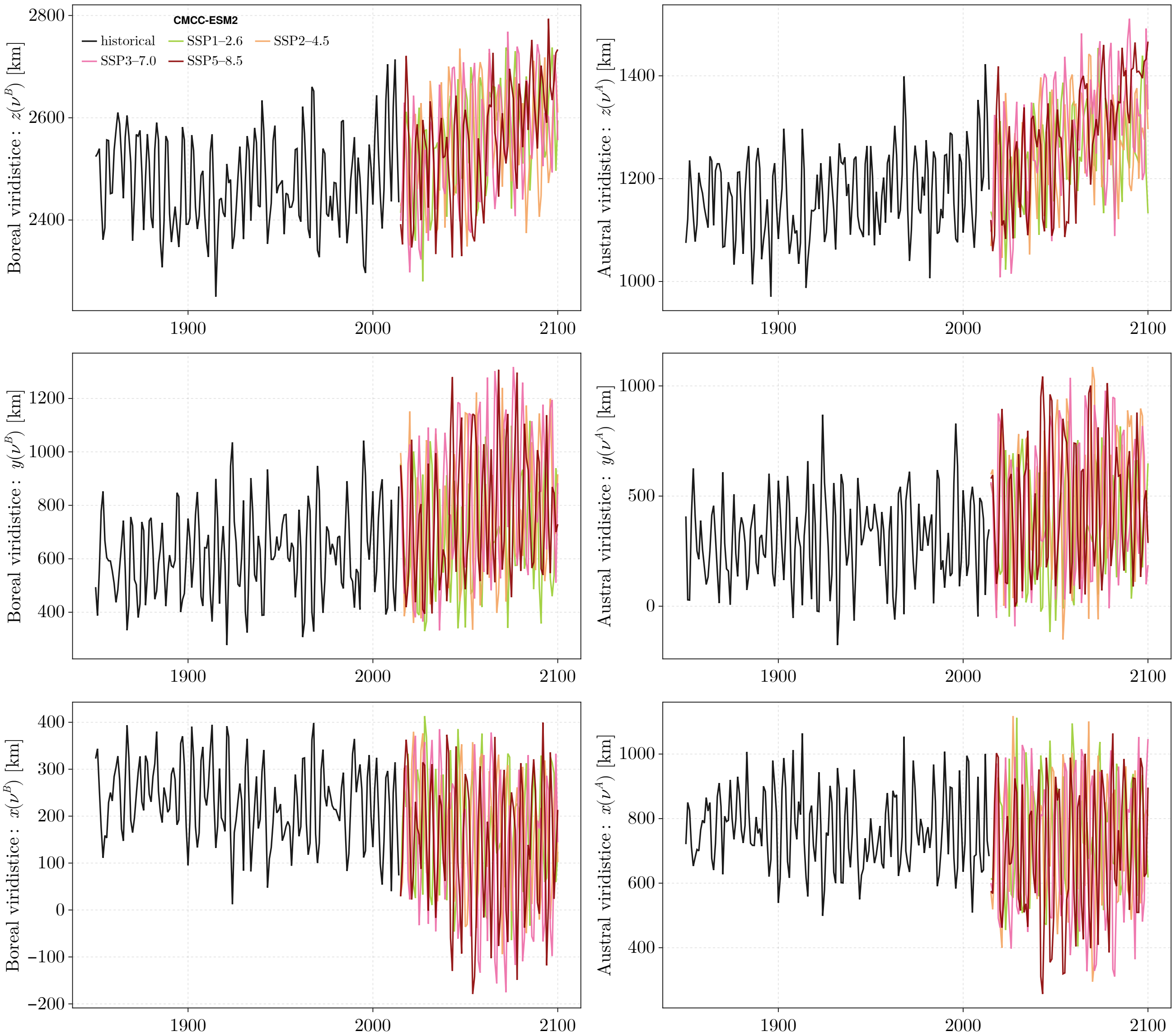

Fig. S41. z, y, and x-coordinates (top to bottom) of the green wave centroid trajectory positions during boreal and austral (left to right) viridistices ($z(\nu^A_{\text{year}})$ and $z(\nu^B_{\text{year}})$) in historical simulations and four Shared Socioeconomic Pathways (SSP) scenarios of the CMIP6 run by the CMCC-ESM2 model. If there are several model runs available, the mean ± 1SD is shown.

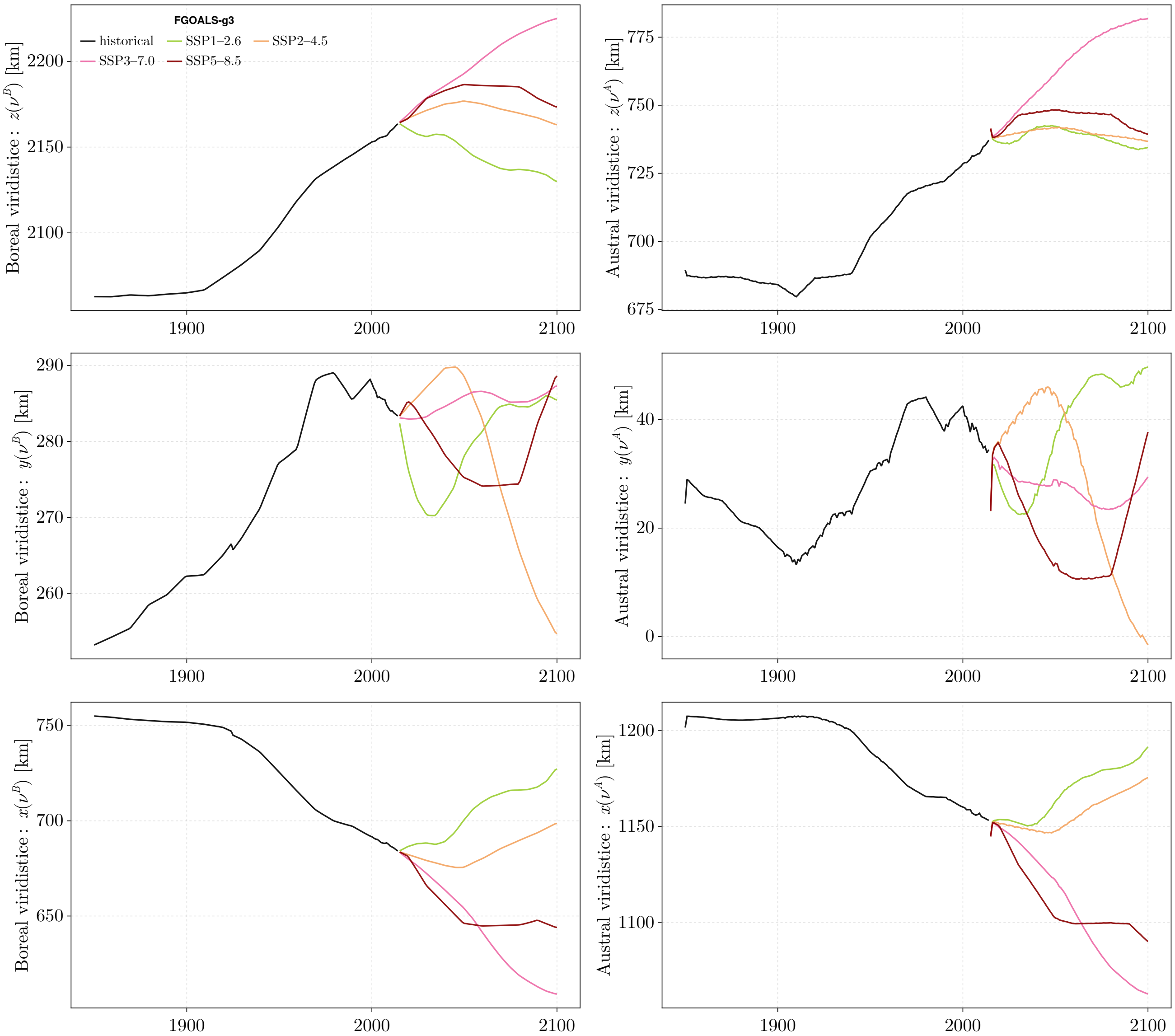

Fig. S42. z, y, and x-coordinates (top to bottom) of the green wave centroid trajectory positions during boreal and austral (left to right) viridistices ($z(\nu^A_{\text{year}})$ and $z(\nu^B_{\text{year}})$) in historical simulations and four Shared Socioeconomic Pathways (SSP) scenarios of the CMIP6 run by the FGOALS-g3 model. If there are several model runs available, the mean ± 1SD is shown.

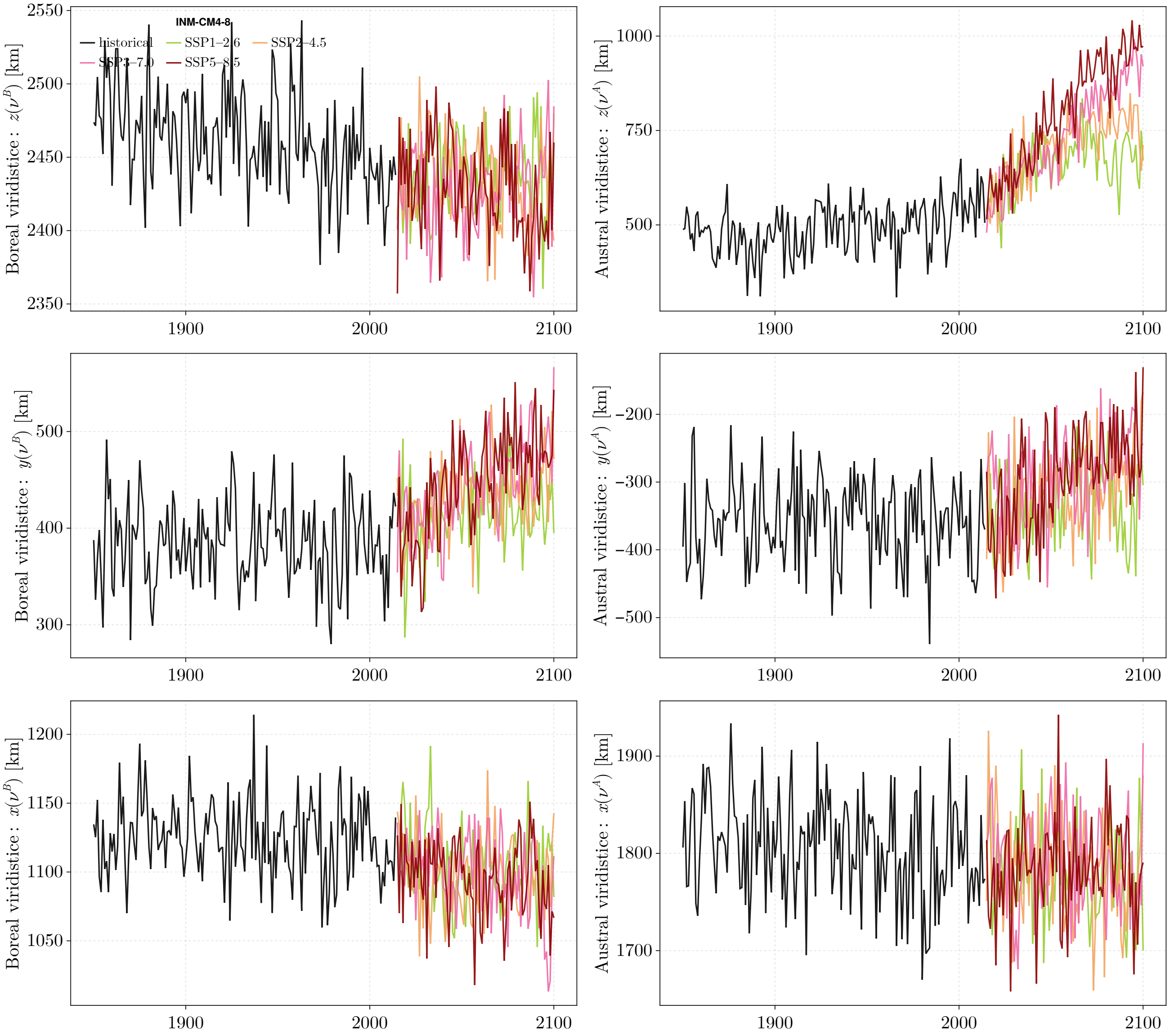

Fig. S43. z, y, and x-coordinates (top to bottom) of the green wave centroid trajectory positions during boreal and austral (left to right) viridistices ($z(\nu^A_{\text{year}})$ and $z(\nu^B_{\text{year}})$) in historical simulations and four Shared Socioeconomic Pathways (SSP) scenarios of the CMIP6 run by the INM-CM4-8 model. If there are several model runs available, the mean ± 1SD is shown.

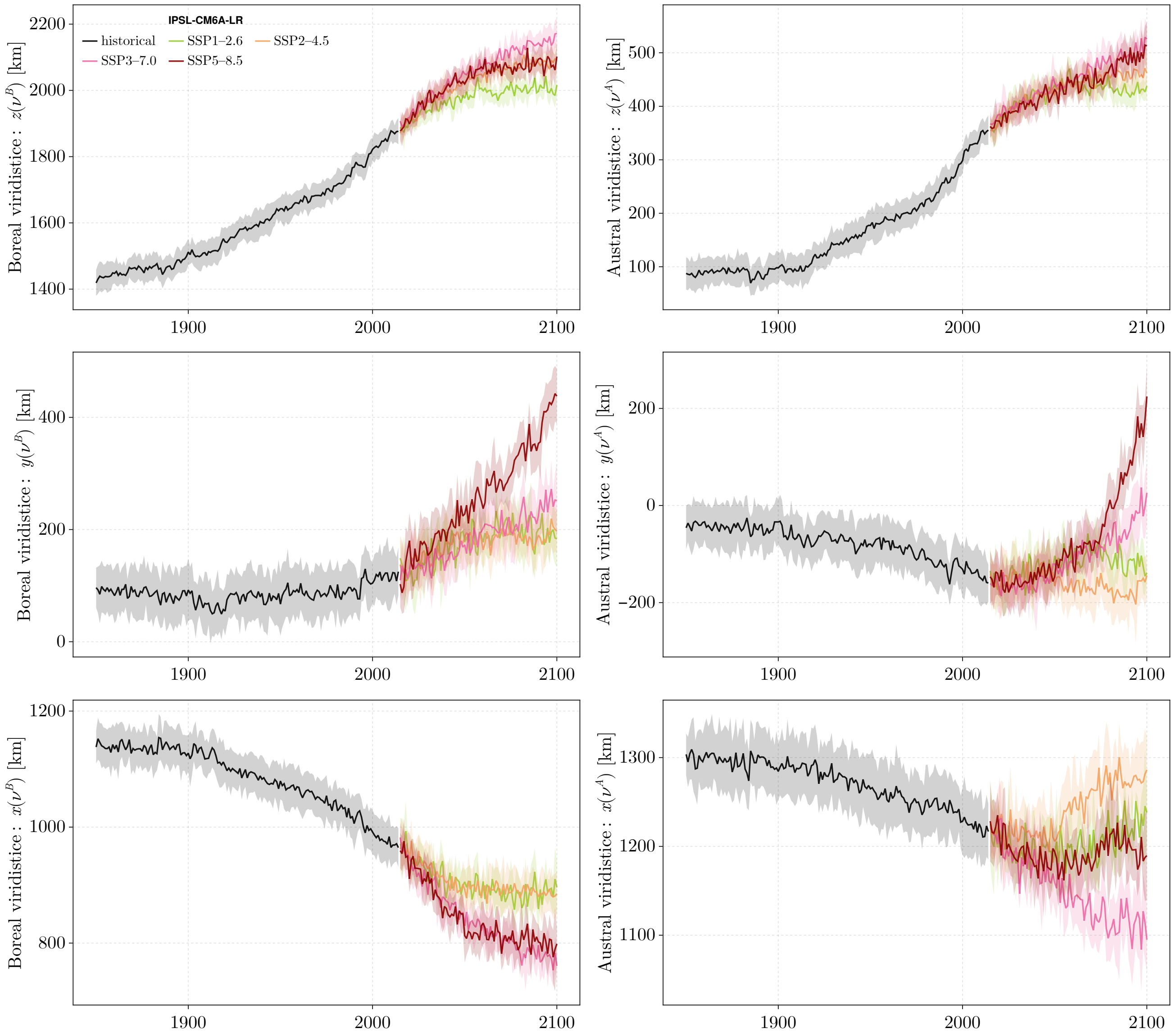

Fig. S44. z, y, and x-coordinates (top to bottom) of the green wave centroid trajectory positions during boreal and austral (left to right) viridistices ($z(\nu^A_{\text{year}})$ and $z(\nu^B_{\text{year}})$) in historical simulations and four Shared Socioeconomic Pathways (SSP) scenarios of the CMIP6 run by the IPSL-CM6A-LR model. If there are several model runs available, the mean ± 1SD is shown.

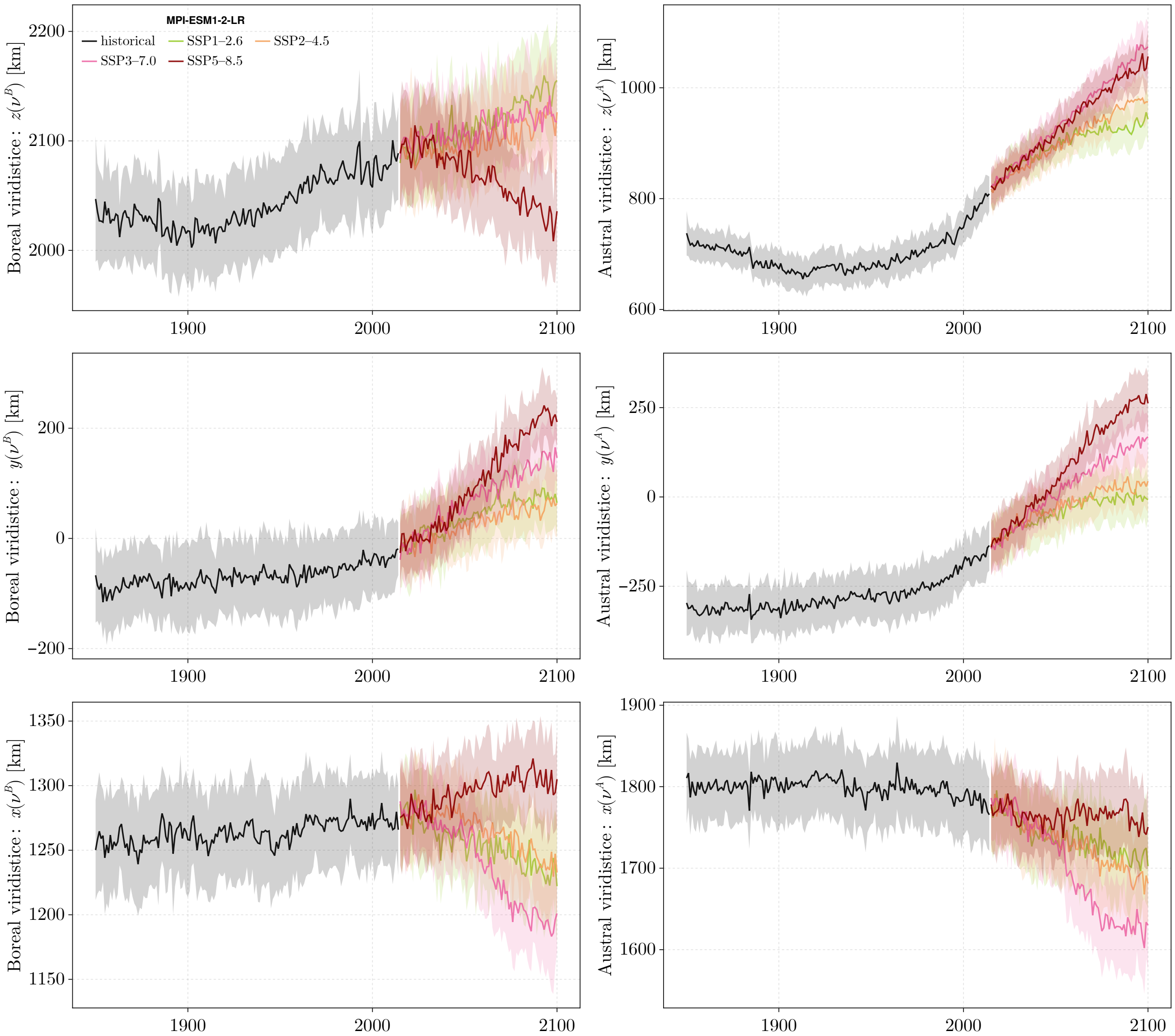

Fig. S45. z, y, and x-coordinates (top to bottom) of the green wave centroid trajectory positions during boreal and austral (left to right) viridistices ($z(\nu^A_{\text{year}})$ and $z(\nu^B_{\text{year}})$) in historical simulations and four Shared Socioeconomic Pathways (SSP) scenarios of the CMIP6 run by the MPI-ESM1-2-LR model. If there are several model runs available, the mean $\pm$1SD is shown.

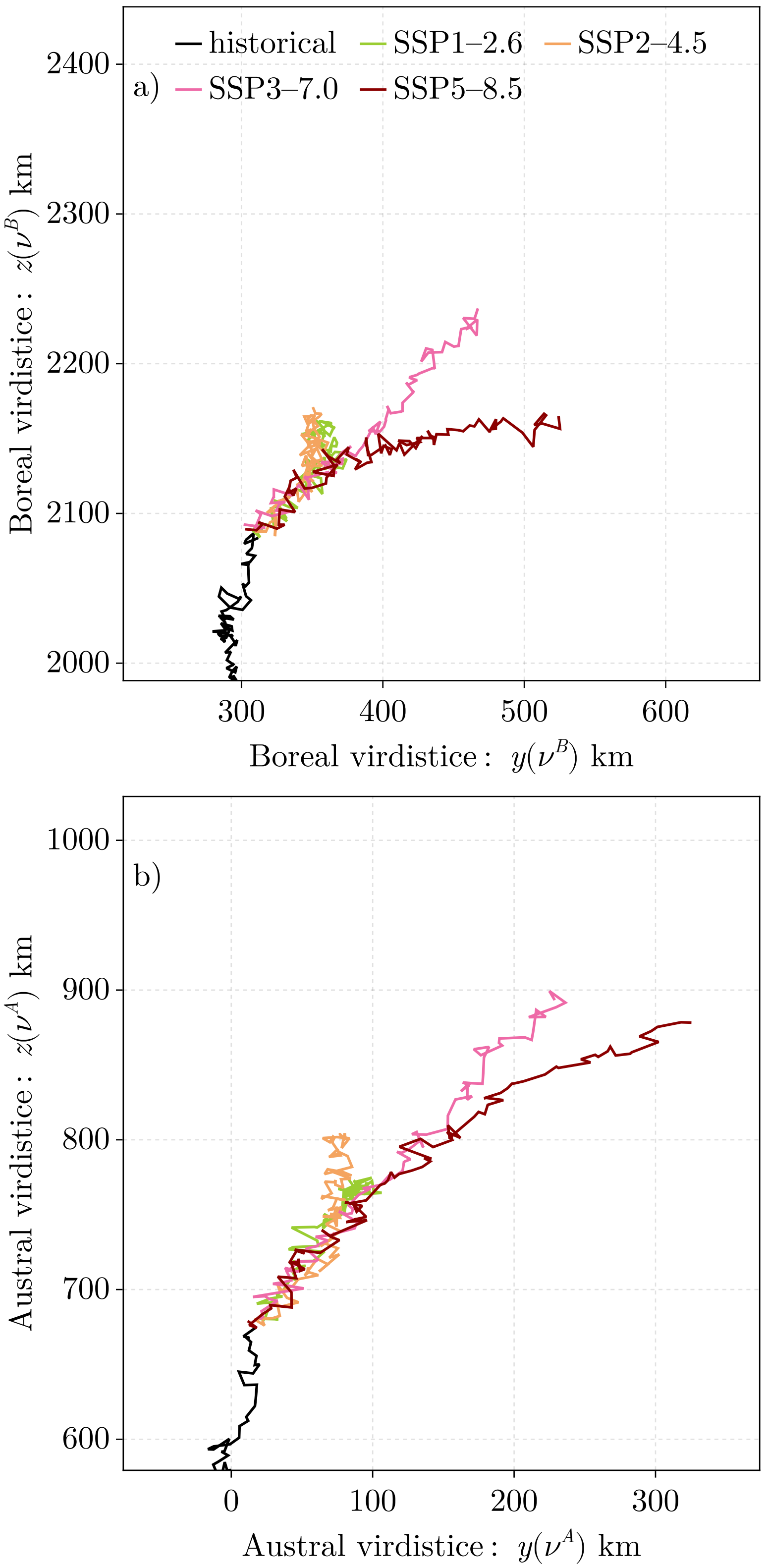

Fig. S46. y versus x-coordinates of the green wave centroid trajectory positions during boreal and austral (left to right) viridistices in historical simulations and four Shared Socioeconomic Pathways (SSP) scenarios for the model ensemble mean.

References

1. S Cao, et al., Spatiotemporally consistent global dataset of the gimms leaf area index (gimms lai4g) from 1982 to 2020. *Earth Syst. Sci. Data Discuss.* **2023**, 1–31 (2023).
2. H Ma, S Liang, Development of the glass 250-m leaf area index product (version 6) from modis data using the bidirectional lstm deep learning model. *Remote. Sens. Environ.* **273**, 112985 (2022).
3. Y Liu, R Liu, JM Chen, Retrospective retrieval of long-term consistent global leaf area index (1981–2011) from combined avhrr and modis data. *J. Geophys. Res. Biogeosciences* **117** (2012).
4. C Schaaf, Z Wang, Modis/terra+aqua brdf/albedo nadir brdf-adjusted ref daily l3 global 0.05deg cmg v061 (2021).
5. JW Rouse, RH Haas, JA Schell, DW Deering, , et al., Monitoring vegetation systems in the great plains with erts. *NASA Spec. Publ* **351**, 309 (1974).
6. G Camps-Valls, et al., A unified vegetation index for quantifying the terrestrial biosphere. *Sci. Adv.* **7** (2021).
7. M Li, et al., Spatiotemporally consistent global dataset of the gimms normalized difference vegetation index (pku gimms ndvi) from 1982 to 2022. *Earth Syst. Sci. Data* **15**, 4181–4203 (2023).
8. X Li, J Xiao, A global, 0.05-degree product of solar-induced chlorophyll fluorescence derived from oco-2, modis, and reanalysis data. *Remote. Sens.* **11**, 517 (2019).
9. S Nelson*, J.A. Walther*, et al., X-base: the first terrestrial carbon and water flux products from an extended data-driven scaling framework, fluxcom-x. *Biogeosciences* **21**, 5079–5115 (2024).
10. Z Xiao, S Liang, B Jiang, Evaluation of four long time-series global leaf area index products. *Agric. For. Meteorology* **246**, 218–230 (2017).
11. S Plummer, et al., The GLOBCARBON initiative global biophysical products for terrestrial carbon studies. *IEEE Int. Geoscience Remote. Sens. Symposium* pp. 2408–2411 (2007).
12. MD Mahecha, et al., Earth system data cubes unravel global multivariate dynamics. *Earth Syst. Dyn.* **11**, 201–234 (2020).
13. D Montero, et al., Earth system data cubes: Avenues for advancing earth system research. *Environ. Data Sci.* (2025).
14. V Eyring, et al., Overview of the coupled model intercomparison project phase 6 (cmip6) experimental design and organization. *Geoscientific Model. Dev.* **9**, 1937–1958 (2016).
15. L Brunner, M Hauser, R Lorenz, U Beyerle, *The ETH Zurich CMIP6 next generation archive: technical documentation* (ETH Zurich, Institute for Atmospheric and Climate Science, Universitätstrasse 16, 8092 Zurich, Switzerland), (2020).
